# Genetic Characterization and Phylogeographic Analysis of the First H13N6 Avian Influenza Virus Isolated from Vega Gull in South Korea

**DOI:** 10.3390/v16020285

**Published:** 2024-02-12

**Authors:** Rochelle A. Flores, Paula Leona T. Cammayo-Fletcher, Binh T. Nguyen, Andrea Gail M. Villavicencio, Seung Yun Lee, Yongwoo Son, Jae-Hoon Kim, Kwang Il Park, Won Gi Yoo, Yeung Bae Jin, Wongi Min, Woo H. Kim

**Affiliations:** 1 Institute of Animal Medicine, College of Veterinary Medicine, Gyeongsang National University, Jinju 52828, Gyeongnam, Republic of Korea; floresrochellea@gmail.com (R.A.F.); cammayopaula@gmail.com (P.L.T.C.-F.); thanhbinhcnty@gmail.com (B.T.N.); andreagail.dvm@gmail.com (A.G.M.V.); seungyun0218@gnu.ac.kr (S.Y.L.); ywson@gnu.ac.kr (Y.S.); kipark@gnu.ac.kr (K.I.P.); ybjin@gnu.ac.kr (Y.B.J.); wongimin@gnu.ac.kr (W.M.); 2 National Park Research Institute, Korean National Park Service, Wonju 26441, Gangwon, Republic of Korea; jh-k@knps.or.kr; 3 Department of Parasitology and Tropical Medicine, College of Medicine, Gyeongsang National University, Jinju 52727, Gyeongnam, Republic of Korea; wgyoo@gnu.ac.kr

**Keywords:** avian, avian influenza, LPAI, gull, South Korea

## Abstract

Avian influenza virus (AIV) is a pathogen with zoonotic and pandemic potential. Migratory birds are natural reservoirs of all known subtypes of AIVs, except for H17N10 and H18N11, and they have been implicated in previous highly pathogenic avian influenza outbreaks worldwide. This study identified and characterized the first isolate of the H13N6 subtype from a Vega gull (*Larus vegae mongolicus*) in South Korea. The amino acid sequence of hemagglutinin gene showed a low pathogenic AIV subtype and various amino acid substitutions were found in the sequence compared to the reference sequence and known H13 isolates. High sequence homology with other H13N6 isolates was found in HA, NA, PB1, and PA genes, but not for PB2, NP, M, and NS genes. Interestingly, various point amino acid mutations were found on all gene segments, and some are linked to an increased binding to human-type receptors, resistance to antivirals, and virulence. Evolutionary and phylogenetic analyses showed that all gene segments are gull-adapted, with a phylogeographic origin of mostly Eurasian, except for PB2, PA, and M. Findings from this study support the evidence that reassortment of AIVs continuously occurs in nature, and migratory birds are vital in the intercontinental spread of avian influenza viruses.

## 1. Introduction

Worldwide, the avian influenza virus (AIV) is an economically significant pathogen in the poultry industry that causes infectious viral diseases that can potentially cause zoonotic and pandemic outbreaks [1]. AIV is an enveloped virus from the Orthomyxoviridae family with a single-stranded, negative-sense RNA characterized by a segmented genome fragmented into eight segments to code for more than 11 viral proteins [2]. These proteins are either inside the lipid envelope (PB1, PB2, PA, NP, M1, NS1, and NEP) or embedded in the envelope (M2, HA, and NA), and each protein functions individually or forms complexes with a structural, nonstructural, or regulatory function during the virus’ life cycle [3,4]. The viral subtypes of AIVs are characterized based on the antigenic properties of the major surface glycoproteins, hemagglutinin (HA), and neuraminidase (NA), and a total of 18 HA and 11 NA subtypes have been identified so far. HA 1-16 and NA 1-9 were identified in avian species, mostly in wild birds, in different combinations, while H17-18 and N10-11 were exclusively identified in bats [2,4,5,6,7].

AIVs are further classified as low-pathogenic avian influenza (LPAI) or highly pathogenic avian influenza (HPAI) based on their pathogenicity in chickens and the molecular characteristics of the HA surface protein, particularly the presence of a poly-basic proteolytic cleavage site between HA1 and HA2 subunits [8]. Only low-pathogenic avian influenza virus serotypes H5 and H7 have been shown to be precursors of HPAIVs [9]. Wild birds, including ducks, gulls, and shorebirds, are natural reservoirs of AIVs of all known subtypes except H17N10 and H18N11. Unlike infected domestic birds, wild birds often exhibit mild or no clinical symptoms when infected [10]. Migratory waterfowls are critical in introducing AIVs to foreign reservoirs during their long-distance migrations along the avian flyway [11]. Although intercontinental reassortment involving the exchange of single or multiple viral segments is rare, it is relatively high among H13 and H16 AIV subtypes, which is believed to be due to the long-distance migration of gull species and overlapping migratory flyways [12,13].

South Korea belongs to the East Asian–Australian flyway, one of the major flight paths in the annual bird migration that supports the most significant migratory bird population, especially in the fall–winter period, for breeding and wintering. The first outbreaks of LPAI and HPAI were recorded in 1996 and 2003, respectively [14]. Both H5 and H7 AIV subtypes have been detected in South Korea. However, all HPAIVs identified to date in South Korea are H5-subtype viruses, which are genetically distant from each other. Additionally, the HPAI outbreaks of novel H5Nx viruses are closely related to migratory birds, and a genetic analysis of the 2016 novel H5N6 HPAIV in South Korea isolated from a migratory bird showed that the newly reassorted virus resulted from different subtypes of H5N6, H4N2, and H1N1 [15].

The genetic characterization of AIVs provides fundamental information that can aid in understanding the mechanisms of viral transmission and mutations to improve surveillance and to determine potential spillover risks to important poultry species that are highly susceptible to AIVs. This study presents the isolation and genetic characterization of the first H13N6 isolate in South Korea. It aims to elucidate the relationship and origin of the H13N6 South Korean isolate to present the patterns of transmission and describe the possible directionality of the virus’ evolution.

## 2. Materials and Methods

### 2.1. Sample Collection and Influenza Virus Preliminary Screening

Wild bird carcass samples were collected in 2021 between February and August from three different collection sites, habitats of wild birds (Heuksan Island, Tongyeong Area and Taean Peninsula), in South Korea. A total of 91 wild bird carcasses from 44 individual avian species were collected and sent to the Veterinary Infectious Disease Laboratory in Gyeongsang National University in a refrigerated and sealed container for disease diagnosis. Sample preparation and pre-processing were carried out as described [16]. Initially, the cloacal swab and tracheal swab were collected and suspended in phosphate-buffered saline (PBS) with antibiotics (1% gentamicin), and the RNA was extracted using QIAamp Viral RNA (Qiagen, Hilden, Germany) following the manufacturer’s protocol. Following the standard protocol for AIV surveillance in South Korea, a preliminary diagnosis to detect influenza virus with one-step real-time RT-PCR was performed using influenza-specific primers and probes targeting M, H5, and H7 genes [17].

### 2.2. Isolation of the Virus

The detection and isolation of AIV was performed on a real-time RT-PCR M gene-positive sample, as described, with modification [8]. In brief, the resuspended swab sample in PBS, supplemented with an antibiotic, was centrifuged (3000 rpm; 10 min), and the supernatant was filtered (0.45 µm) and inoculated into specific-pathogen-free (SPF) embryonated chicken eggs (9–11 days old), before being incubated at 37 °C for 72 h–120 h. After incubation, allantoic fluid was harvested and a hemagglutination assay was performed, then another screening of M gene detection was carried out using real-time RT-PCR, as described above.

### 2.3. Viral RNA Extraction and Sequencing

Viral RNA (vRNA) from the HA- and M-gene-positive allantoic fluid sample was extracted using QIAamp Viral RNA (Qiagen, Hilden, Germany) following the manufacturer’s instructions. The cDNA synthesis of the vRNA was carried out using Quantitect Reverse Transcription Kit (Qiagen, Hilden, Germany) with modification using uni-12 primers. Subsequently, amplification of the full-length sequences of the eight viral genomic segments was conducted using influenza-specific primers in a thermal cycler PCR, following the PCR conditions described previously (Table S1). The amplicons of each gene segment were visualized by gel electrophoresis and purified using Favorgen Gel Purification Kit (Favorgen Biotech Corp., Pingtung, Taiwan). Purified PCR products were then sent for sequencing.

### 2.4. Homology and Amino Acid Mutation Analysis

The homology of the gene sequences of the isolate (SKH13N6) was compared to the sequences in the NCBI and GISAID databases using BLAST. The identity and similarity percentages of the HA and NA sequences compared to other H13N6 isolates were assessed with EMBOSS Needle pairwise alignment (https://www.ebi.ac.uk/Tools/psa/emboss_needle/) accessed on 24 May 2023. The amino acid sequence of SKH13N6 was determined using ORF (https://www.ncbi.nlm.nih.gov/orffinder/) accessed on 8 June 2023 and the amino acid variant and mutation analyses to the closest gene reference sequences of the influenza virus were performed in FluSurver (https://flusurver.bii.a-star.edu.sg/) accessed on 17 November 2023. FluSurver is a tool available in GISAID, which is used to identify and interpret mutations in influenza sequences. Additionally, the identification of new amino acid variants in SKH13N6 compared to the H13N6 reference (MDH13N6, A/gull/Maryland/704/1977(H13N6)) and other known H13N6 isolates was carried out using DNAStar MegAlign Software, and the relative number of amino acid mutations for each gene was calculated. All sequences used for amino acid mutation analysis were downloaded and retrieved from the NCBI Influenza Virus Database (https://www.ncbi.nlm.nih.gov/genomes/FLU/Database/nph-select.cgi) and GenBank (https://www.ncbi.nlm.nih.gov/genbank/) accessed on 8 June 2023. Sequences of SKH13N6 genes were deposited in GenBank database (accession numbers: OR037484-OR037491).

### 2.5. Dataset and Data Preparation for Phylogenetic Analysis

All the sequences used for downstream phylogenetic analysis were downloaded and retrieved from the NCBI Influenza Virus Database. Sequences for each gene segment were filtered following a defined search set for each gene segment using all available full-length sequences of HA (H13Nx isolates), NA (HxN6 isolates), and internal genes (H13Nx isolates). The retrieved sequences were further downsampled by representing all groups of identical sequences in each dataset using the oldest isolate in the group. In each data set, nucleotide sequences of the isolates were aligned using MUSCLE and trimmed using only the coding region in MEGA 11 [18]. A maximum-likelihood (ML) method following the GTR- gamma substitution model with 1000-replicates bootstrap was constructed using MEGA 11, and the relationship between genetic divergence and time (temporal signal) was examined using TempEst v1.5.3 [19]. The final dataset used for the evolutionary origin and phylogeographic analyses of PB2 (*n* = 201), PB1 (*n* = 210), PA (*n* = 232), HA (*n* = 84), NP (*n* = 156), NA (*n* = 158), M (*n* = 164), and NS (*n* = 99) is summarized in Tables S2–S9.

### 2.6. Evolutionary and Phylogeographic Analysis

An evolutionary analysis, including the time to the most recent common ancestors (TMRCA) and the phylogenetic origin of SKH13N6 gene segments, was carried out using the Bayesian Markov Chain Monte Carlo (MCMC) method in BEAST v1.10.4 [20]. In all simulations, analyses were reconstructed following a generalized, time-reversible (GTR) substitution with a gamma site heterogeneity model (Yang96 model) [21], uncorrelated relaxed lognormal clock [22], and Bayesian skyline coalescent tree prior [23,24]. Two independent MCMC analyses were carried out on all gene segments with 200 million generations (PA, HA, NP, NA, M, and NS) and 400 million generations (PB2 and PB1), sampled every 10,000 runs. All MCMC analyses were evaluated for parameter convergence and effective sample size (ESS, >200) in Tracer v1.7.2 [25], and each independent run was combined using LogCombiner v1.10.4 with at least 10% burn-in removed. Post analysis to summarize the results of the maximum clade credibility (MCC) tree was created using TreeAnnotator v1.10.4 and visualized using FigTree v1.4.4 (http://tree.bio.ed.ac.uk/software/figtree/) accessed on 3 November 2023. Discrete traits, including geographic location (Asia, Europe, North America, Oceania, South America) and host species or source (Order *Anseriformes* and *Galliformes*, Environment, Gull, Mammal, Shorebird, Other *Charadriiformes*), were also included in the analysis (Tables S2–S9).

## 3. Results

### 3.1. Surveillance and AIV Subtype

**Figure 1:**
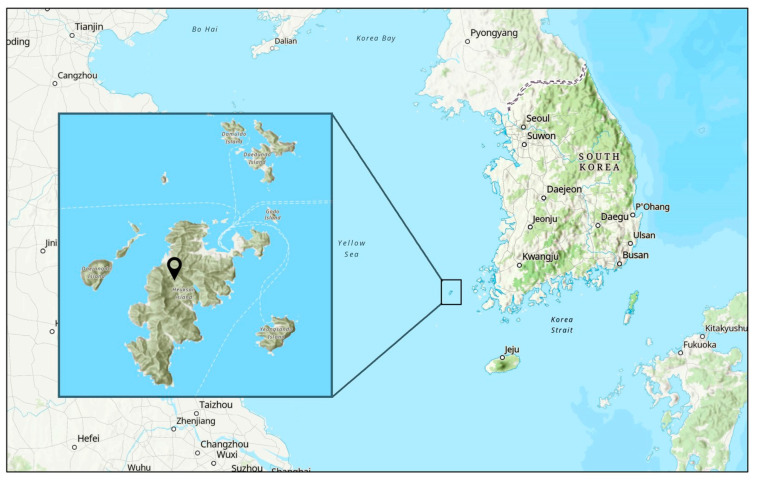
Geographical location of the sampling site in South Korea. The map image was retrieved from maps.arcgis.com. The inset shows Heuksan Island.

Of the 91 wild bird carcass samples collected from February to August 2021, 1 was positive for AIV, representing a 1.1% prevalence. The AIV was isolated from the carcass of a Vega gull found in Heuksan Island (34.6707° N 125.42° E), Jeollanamdo, South Korea (Figure 1). The sequencing results for the HA and NA genes of the isolated AIV showed an H13N6 subtype, which was identified as A/Vega gull/South Korea/GNU54/2021 following the nomenclature of AIVs. In this study, the South Korean H13N6 isolate is referred to as SKH13N6.

### 3.2. HA Gene Exhibits LPAI with Variable Amino Acid Substitutions

The highest homology of the HA nucleotide sequence was with A/common_gull/Poland/MW241/2011(H13N6) at 95.42% (Table 1), while 87% homology was found with the H13N6 reference isolate, MDH13N6 (A/gull/Maryland/704/1977(H13N6)) (Table 2). The percent identity to other H13N6 isolates from various hosts was 74.4–91.69% to ducks and swan, 74.1–93.4% to shorebirds and curlews, and 73.9–95.22% to gulls. Likewise, the amino acid’s identity-based similarities with reference MDH13N6, Anatidae (ducks and swan), other wild birds (shorebirds, curlew), and gulls were 92.6%, 82.7–94.5%, 85–94.9%, and 82.5–95.2%, respectively (Table 2). The deduced HA amino acid sequence of the SKH13N6 isolate showed that the HA gene is classified in Group 1, along with the H13 influenza virus and the Sialic acid (SA) receptor binding site.

**Figure 2:**
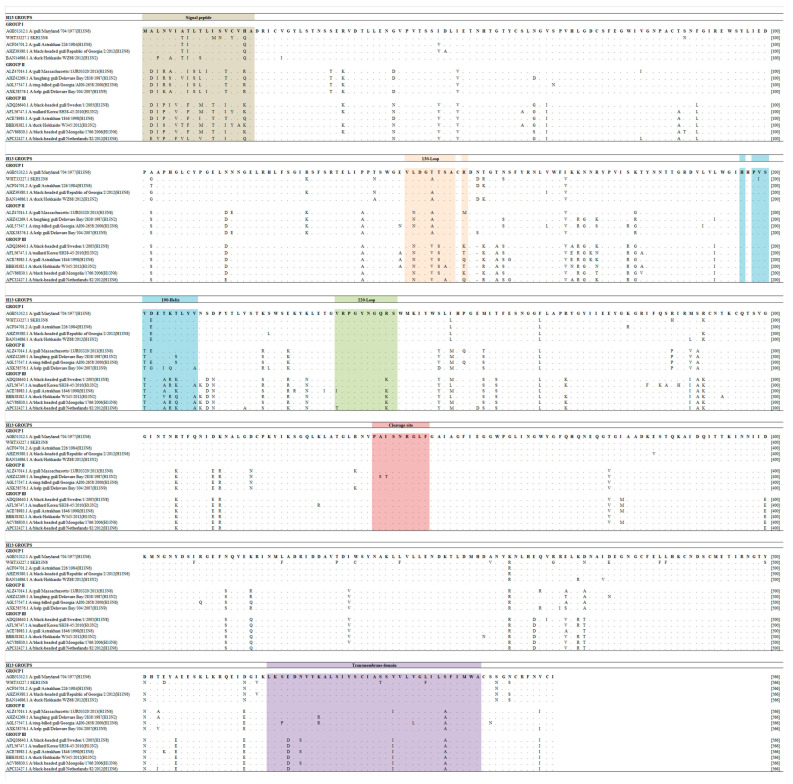
Clustal alignment of SKH13N6 HA sequence compared to other H13 isolate sequences. Amino acid alignment and comparison with SKH13N6 and other representative sequences of H13 groups was performed using MUSCLE. The signal peptide sequence, SA receptor binding sites (130 loop, 190 helix, and 220 loop), cleavage site, and transmembrane domain are highlighted in Khakhi, light orange, sky blue, light green, rose and lavender color, respectively, following the H3 numbering system. The H13N6 reference sequence (MDH13N6) is shown on the top of the line. The accession number of each isolate is written before the isolate ID.

**Figure 3:**
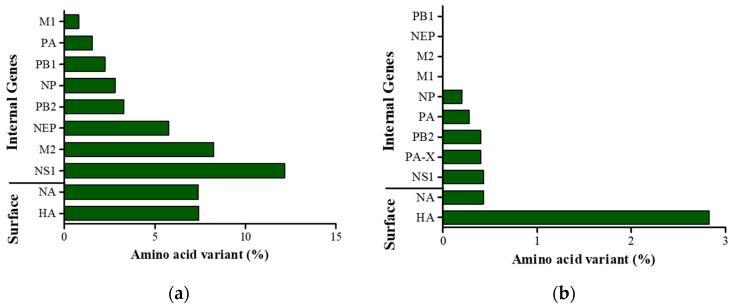
Relative number of amino acid variants in SKH13N6 gene segments. (**a**) Percentage of amino acid variants in SKH13N6 gene sequences compared to the gene sequences of H13N6 reference isolate MDH13N6. (**b**) Percentage of amino acid variants of gene sequences found only in the SKH13N6 isolate compared to known H13N6 isolates of Eurasian and North American lineage. Bar represents the mutation for each segment relative to the full amino acid length of the sequence.

The 130-loop, 190 helix, and 220-loop were generally conserved, and the amino acid motif, PAISNR/GLF, at the cleavage site was consistent with those of other LPAIVs. However, a point amino acid substitution in the transmembrane domain was found in positions 539 and 546, and these amino acid variants are different from the reference MDH13N6 and other H13 sequences (Figure 2). Generally, the HA gene of SKH13N6 isolate had no amino acid deletions. However, a 7.42% (42/566) amino acid substitution at the variable position was observed compared to the reference MDH13N6 (Figure 3a, Table 3), whereas a 2.83% (16/566) unique amino acid substitution was found compared to other known H13N6 isolates (Figure 3b, Table S10). The identification of K193T and G228S amino acid mutations related to their enhanced binding to a human receptor, α2-6, was also found in the isolate (Table 4).

### 3.3. NA Gene Is Highly Adapted to Gull

The nucleotide sequence of the SKH13N6 NA gene shares a percent identity of 87.7%, 76.6–94.9%, 76.6–97.3%, and 77.2–98.33% with the reference MDH13N6, Anatidae, wild birds, and gull sequences, respectively, with the highest homology with the isolate A/Glaucous-Winged Gull/Alaska/20MB02534/2020 (H13N6) (Table 1 and Table 5). Similarly, the amino acid sequence is more gull-adapted, with a 92.6% homology to the reference MDH13N6 and an 86.2–98.7% to known isolates from gulls as compared to the isolates collected from Anatidae (86.4–94.5%) and other wild birds (85.7–98.1%) (Table 5). Compared to the reference MDH13N6, the NA-6 gene of the SKH13N6 isolate has a 7.4% (35/471) amino acid substitution (Figure 3a), with two amino acid deletions at positions 55 and 66 (Table 3). Compared to other H13N6 isolates, a 0.43% unique amino acid substitution was found in the SKH13N6 isolate, particularly in positions 253 and 391 (Figure 3b, Table S10). Additionally, a I117T amino acid mutation was identified in the SKH13N6 isolate previously reported to be related to the resistance against anti-viral drugs, such as oseltamivir or zanamivir (Table 4). 

### 3.4. Internal Gene Sequences with Virulence-Related Mutations

PA and NP genes of SKH13N6 have the highest homology with the H13N6 virus, isolated from gulls, while PB1 has the highest homology with an H13N6 virus from shelduck. Interestingly, PB2, M, and NS genes share the highest homology with an H13N8 gene isolated from gulls (A/*Chroicocephalus_ridibundus*/Belgium/13464/2020) (Table 1). Multiple alignments of the full-length, deduced amino acid sequences of SKH13N6 internal genes and other H13N6 isolates showed new amino acid changes for NP, PA, PB2, PA-X, and NS1 sequences at specific positions representative of a low percentage of amino acid substitution (0.2–0.43%), whereas no new amino acid substitutions were found for PB1, NEP, M1, and M2 genes (Figure 3b; Table S10). On the other hand, when comparing the amino acid sequences of SKH13N6 to H13N6 reference MDH13N6, 0.8–12.2% amino acid changes to different internal gene sequences at different positions were observed, with the NS1 gene having the highest and M1 the lowest rate of amino acid substitutions (Figure 3a). Accordingly, the percent identity with the reference MDH13N6 was generally high for M1 (99.2%), PA (98.5%), PB1 (97.8%), NP (97.2%), PB2 (96.7), NEP (94.2%), and M2 (91.8%), while it was moderately high for NS1 (87.8%) (Table S11). Using FluSurver, several amino acid mutations on the internal genes were also found compared to the closest reference for the segment gene in the database, with the highest number of mutations found in NS1 (27/230). A number of mutations were also found for M2 (6/97), PB2 (18/759), PB1 (14/757), and PA (8/716) (Table 3). Amino acid mutations found to be related to drug resistance and virulence in mammalian cells and mice were also determined on the internal genes of SKH13N6. No amino acid variants related to drug resistance were found on the SKH13N6 isolate M2 gene. However, amino acid mutations related to increased virulence were found for NS1, M1, NP, and all polymerase genes (PA, PB1, and PB2). Specifically, two amino acid mutations were found in NS1 (P24S, V149A), NP (M105V, A184K), and M1 (N30D, T215A), whereas three, six, and eight amino acid mutations were present for PB1 (D3V, D622G, H436Y), PA (S37A, P190S, N383D, N409S, I550L, K615N), and PB2 (L89V, I292V, G309D, T339K, R477G, I495V, I504V, A676T) genes, respectively (Table 4).

### 3.5. Phylogenetics and Origin of SKH13N6

**Figure 4:**
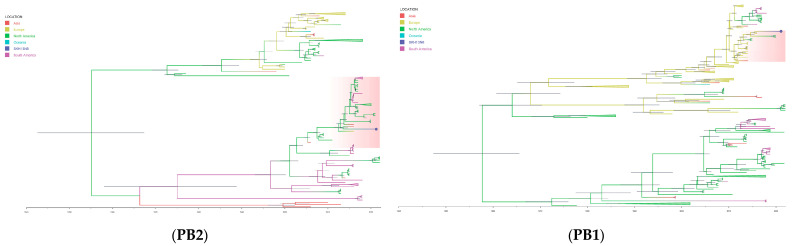

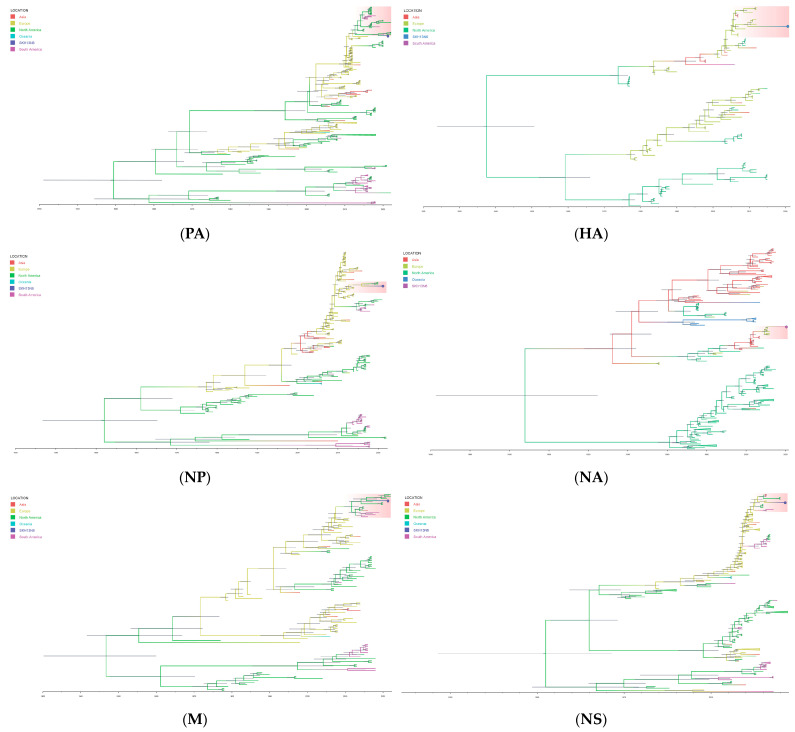
Time-scaled phylogeographic tree of SKH13N6. The branches of the maximum clade credibility (MCC) tree of the gene segments are colored according to the origin of the virus isolates. The geographic location is classified as Asia, Europe, North America, Oceania, and South America. The cluster of SKH13N6 is highlighted and the isolate is indicated with a circle (●). The scale bars indicate years, and the node bars represent 95% highest posterior density (HPD) from the dates of the most recent common ancestors (TMRCAs). The full lengths of the coding sequence for all genes were used in the analysis.

The average date of the most common ancestor (TMRCA) of SKH13N6 surface genes, HA and NA, ranges from 2009.5347 (95% HDP = 2008.5285-2010.4524; posterior probability = 1.0) and 2010.5671 (95% HDP = 2009.8727-2010.9990; posterior probability = 0.9963), respectively (Table 6). A phylogeographic analysis of both genes showed that the introduction of the most recent virus ancestor followed a path from Asia to Europe, then from Europe to South Korea (Figure 4). Similarly, the TMRCA of PB1 (2015.4666), NP (2015.8315), and NS (2015.4673) are closely related to each other and occurred within the same time period in Europe (posterior probability = 1.0). Conversely, the phylogeographic origin of PB2, M, and PA gene segments of SKH13N6 shares a common ancestor involving North American avian influenza strains around 2013.4985 (2012.3697-2014.6044), 2015.5910 (2012-0967-2018.3715), and 2018.6772 (2016.9407-2020.1785), respectively (Table 6, Figure 4). For all gene segments, SKH13N6 originated from gulls (Figures S1–S8).

## 4. Discussion

The surveillance of circulating influenza virus among wild birds is vital since migratory birds are considered natural reservoirs of the Influenza A viruses, and several studies have linked the role of wild birds in virus transmission to domestic poultry, including related HPAI outbreaks [15,44,45]. Since the first HPAI outbreak in South Korea in 2003, active nationwide surveillance of AIVs has been implemented. This has since resulted in the isolation of various AIV subtypes including not only HPAIVs but LPAIVs from wild birds, ducks, and poultry from live bird markets, farms, and wild bird habitats [5,8,46]. In the present study, the first H13N6 subtype in South Korea was identified and isolated from a carcass of a Vega gull found on Heuksan Island. 

HA analysis of the SKH13N6 isolate showed that the cleavage site has an LPAI amino acid motif consistent with other H13 HA, AISNR/GLFG [10]. In a previous study, H13 subtypes were phylogenetically divided into three groups (Groups I, II, and III). Group I was more antigenically related to Group II but distinct from Group III [47]. The isolate, SKH13N6, identified in this study is classified into Group I H13, along with the reference, MDH13N6. Following the results of the antigenic analysis of Wang et al., its inclusion in the H13 group suggests that, since SKH13N6 belongs to Group I, SKH13N6 has closer antigenicity to A/Laughing gull/Delaware Bay/2838/1987(H13N2) and A/Red Knot/Delaware Bay/424/2007(H13N9), rather than A/sanderling/Delaware Bay/224/2006(H13N9) and A/Duck/Hokkaido/W345/2012(H13N2) [47]. Several amino acid mutations or variants on the isolate were also found compared to the H13N6 reference (MDH13N6) and other H13 sequences retrieved from the NCBI Influenza Database. As one of the major viral antigenic determinants of AIVs, point mutations on the HA gene could suggest that the virus may have undergone antigenic drift [48]. Although classified as LPAIV, K193T and G228S mutations were present in the SKH13N6 isolate. The mutation K193T was linked to the enhanced binding of ferret-transmissible H5N1 to the α2-6 glycan receptor, the receptor binding preference of human-adapted influenza virus [26]. On the other hand, the G228S mutation has been found to increase the binding of AIV H4N6 and H5N1 to the α2-6 glycan receptor, suggesting the adaptation of AIVs to human receptors [36,49]. Similarly, AIV H1N2 in mice showed increased virulence and viral replication in mammalian cells due to this mutation [50].

Although at a lower percentage, several point mutations and substitutions were also observed in NA and all internal genes of SKH13N6 compared to the reference sequences in FluSurver, MDH13N6, and other H13 isolates. While no antiviral-resistant amino acid mutation was found in the M2 gene of the isolate, a point mutation of I117T was present in the SKH13N6 NA gene segment. This mutation was previously reported in the HPAIV H5N1 isolate in India and was found to be related to the reduced susceptibility for oseltamivir and zanamivir shown by the NA inhibition assays [28]. Other markers related to antiviral drug resistance were not present on the SKH13N6, and these findings agree with a previous report on the NA genes of other AIVs collected from wild birds in South Korea [8].

An analysis of the internal genes NS1, NP, and M1 of the SKH13N6 isolate also show several amino acid mutations previously reported to be associated with increased virulence in mice and chickens. The single amino acid mutation of P42S in the NS1 gene of H5N1 (A/Duck/Guangxi/12/03) was found to be related to the increased virulence of the virus in mice, while the V149A mutation in H5N1 (A/Goose/Guangdong/1/96) showed increased virulence in chickens, along with a reduced interferon production in chicken embryo fibroblasts (CEFs) [29,31]. Another study with H5N1 demonstrated that the presence of the valine at position 105 (105V) and Lysine (K) at position 184 (184K) in the NP gene resulted in increased pathogenicity in chickens, and 105V may be one of the determinants for AIV’s adaptation from ducks to chicken [32,33]. Using reverse genetics on H5N1 HPAIVs (A/duck/Guangxi/53/2002; A/duck/Fujian/01/2002), Fan et al. (2009) demonstrated that the amino residues 30D and 215A in the M1 protein contribute to the increased pathogenicity of AIVs in mice [38]. 

Compared to the other internal segments of the SKH13N6 isolate, the majority of the amino acid mutations related to virulence were found on the polymerase genes (PA, PB1, and PB2). These mutations were previously found to be related to the increased polymerase activity and enhanced replication in mammalian and avian cells, and contributed to the increased virulence in mice, ducks, and ferrets [34,35,36,37,39,41,42,43,51]. A variety of minor changes in the amino acid components of PB1, PB2, and PA enhance the virulence of the influenza A virus and each virulence-enhancing mutation relates to increased polymerase activity [37]. Likewise, specific mutations in the viral polymerase increase the activity of HPAI in mammalian cells, suggesting its role in mediating the adaptation of AIVs to mammalian hosts [52]. A similar report on virulent H7N3 also identified PB2 and PA polymerase genes as major determinants of AIV pathogenicity in mammalian hosts [35]. In the SKH13N6 isolate, the majority of the amino acid mutations related to virulence were found in PB2. PB2 is a major determinant of influenza virus host range and replication efficiency in mammalian hosts, particularly position 627, with the glutamic acid (E) and lysine (K) found in AIV and the human influenza virus, respectively [53,54]. The mutation E627K was not found in the PB2 gene of the SKH13N6 isolate, but other markers relating to increased virulence and adaptability to the mammalian host were present in the isolate, suggesting that the PB2 gene continues to evolve, developing strategies for host adaptations.

Recent reports on these related amino acid mutations were also found in isolates from wild birds of different subtypes, isolated in South Korea, HPAI H5N1, isolated from bald eagles and chickens in Canada, and the H7N7 virus, isolated from wild birds in China [8,30,55,56]. Overall, the results from this report and from previous reports suggest that it is necessary to routinely monitor not just the surface-protein-related genes but also the internal genes of AIVs, as mutations on these segments can occur, and some have been associated with increased virulence, drug resistance, and host adaptations. Understanding this will provide insight into the evolution and adaptation of the virus, particularly to its host, and will be critical for risk assessment.

The homology of the gene segments is highly similarly to those for gulls, except for PB1, which is closely related to shelduck isolate, the last H13N6 isolate reported in Asia until this report. This finding regarding the gull-adapted internal genes and duck-related PB1 agrees with a previous report on H13N8 [57]. Although H13, along with H16, naturally evolved as a gull-adapted AIV with high prevalence in juvenile and adult gulls in the breeding site and during the spring and/or fall migration, respectively, the isolation of the H13 subtype in shore birds and Anseriformes, such as ducks and geese, has also been reported [58,59]. Ducks sharing the same habitat could potentially increase the risk of virus reassortment and cross-species transmission [57]. A recent analysis of the direction of spillover for all gene segments found that the majority of cases moved from ducks to gulls [60].

South Korea is part of the East-Asian Australasian flyway, and it is the wintering site for wintering migratory birds from the Order *Anseriformes* and the resting site of *Charadriiformes* migrating to Australia [61]. This is the first H13N6 AIV isolated in South Korea, and the genetic characterization results showed that, although classified as LPAI, various degree of mutations on the gene segments were found, in addition to the high homology of some with non-H13N6 and non-gull isolates. To elucidate these findings, a time-scaled phylogenetic analysis was performed of the different gene segments to determine the origin of the isolate and understand the viral evolution of the new isolate. A phylogeographic analysis of the gene segments of SKH13N6 isolate showed that while the majority of the gene segments shared a Eurasian origin, multiple occasions of the introduction of viruses of a North American lineage also occurred, suggesting that the SKH13N6 isolate has undergone an intercontinental mixing of the gene pool and reassorted. Moreover, the TMRCA results show that the internal genes found in this isolate have a more recent ancestor compared to the HA and NA genes, suggesting that the H13N6 circulating in Eurasia is continuously undergoing mutations. Although SKH13N6 genes are gull-adapted, a few H13 viruses were occasionally detected from other hosts, which were closely clustered with SKH13N6, which could explain the high degree of homology found with non-gull hosts. Similar findings, with mixed lineages from Eurasian, North American gulls, and North American waterfowl lineages, were also discovered in the internal genes of AIVs with H13N2 and H13N6 subtypes [58,62]. These findings reflect the diversity from which this isolate originated, affirming that gulls and waterfowls are vital players in virus reassortment and the movement of migratory birds from different geographical origins along flyways greatly contributes to this occurrence.

Although reassortment is relatively rare between geographically separated gene pools, evidence of an intercontinental reassortment of isolates collected from the order *Charadriiformes* along migratory flyways, particularly in overlapping flyways, has been reported [63,64]. Interestingly, all PB1 genes circulating in North America are of Eurasian lineage [12]. Birds from the order *Charadriiformes*, like shorebirds and gulls, migrate biannually between their breeding and wintering sites in the southern and northern regions [7]. Vega gulls are among the arctic gulls involved in a long-distance migration between their breeding and wintering habitats [65]. The recent 2021/2022 HPAI outbreaks in Europe affected several gull species from the *Laridae* family. A recent report on the pathogenicity and transmissibility of HPAI H5N8 among gulls showed that the virus is highly virulent to naïve gulls, pre-exposure to LPAIV can only partially modulate the disease, and convalescent birds are asymptomatic shedders, suggesting that not only are gulls susceptible to the virus, but they can maintain the virus in a given area or disseminate it over particular distances [66]. The intercontinental mixing of the gene pool observed in this study likely resulted from these characteristics and the nature of gulls. 

## 5. Conclusions

Genetic characterization and the related phylogenetic analysis showed that SKH13N6 isolate is a novel, reassorted H13N6 virus. More importantly, amino acid mutations related to increased receptor binding, drug resistance, and virulence were present in the different gene segments of the isolate. However, the overall significance of all the amino acid mutations and the new amino acid variants found in the isolate are beyond the scope of this study; thus, a risk assessment of these mutations is encouraged for future works. The findings from this study corroborated evidence that reassortment of AIVs continuously occurs in nature, and migratory birds are vital in the intercontinental spread of influenza viruses. Moreover, overlapping flyways are a significant contributing factor in the viral gene flow, making it critical that surveillance efforts include the long-term monitoring of the flyways and migratory patterns of migratory birds. Lastly, it is necessary to continue to identify and characterize isolated influenza viruses, regardless of their pathogenicity, as this helps to provide information that will be crucial in controlling and preventing outbreaks.

## Figures and Tables

**Table 1:** Homology analysis of the nucleotide sequences of SKH13N6 gene segments.

SKH13N6 Accession ID	Gene Segment	Highest Homology Virus ^1^	Accession Number	Identity (%)
OR037491.1	PB2	A/*Chroicocephalus_ridibundus*/Belgium/13464/2020 (H13N8)	EPI1942887	97.85
OR037490.1	PB1	A/shelduck/Ukraine/KT-9-2-11/2016 (H13N6)	MW132947.1	98.15
OR037489.1	PA	A/glaucous-winged gull/Southcentral Alaska/15MB01693/2015 (H13N6)	CY213548.1	96.85
OR037484.1	HA	A/common_gull/Poland/MW241/2011 (H13N6)	EPI2195562	95.42
OR037486.1	NP	A/Armenian gull/Republic of Georgia/2/2012 (H13N2)	CY185350.1	97.19
OR037485.1	NA	A/Glaucous-Winged Gull/Alaska/20MB02534/2020 (H13N6)	MZ015698.1	98.33
OR037487.1	M	A/*Chroicocephalus_ridibundus*/Belgium/13464/2020 (H13N8)	EPI1942893	99.29
OR037488.1	NS	A/*Chroicocephalus_ridibundus*/Belgium/13464/2020 (H13N8)	EPI1942894	98.33

^1^ Full length of the coding DNA sequences of the gene segments used for BLAST in NCBI and GISAID a footer. PB2, polymerase basic 2; PB1, polymerase basic 1; PA, polymerase acidic; HA, hemagglutinin; NP, nucleoprotein; NA, neuraminidase; M, matrix; NS, nonstructural.

**Table 2:** Percent identity and similarity with the SKH13N6 HA gene and other known H13N6 isolates.

Isolate ID	Nucleotide		Amino Acid		
	Accession	Identity (%)	Accession	Identity (%)	Similarity (%)
**Gulls**					
A/Armenian gull/Republic of Georgia/1/2012 (H13N6)	CY185355.1	95.08	AHZ39558.1	91.9	94
A/black headed gull/Mongolia/1766/2006 (H13N6)	GQ907302.1	75.1	ACV86830.1	82.5	91
A/black-headed gull/Republic of Georgia/7/2011 (H13N6)	CY185489.1	91.9	AHZ39225.1	94.5	97
A/black-headed gull/Netherlands/26/2014 (H13N6)	KX978072.1	73.9	APC30994.1	83.2	90.5
A/Glaucous-Winged Gull/Alaska/20MB02831/2020 (H13N6)	MZ015629.1	73.9	QUE37731.1	83.7	91
A/Glaucous-Winged Gull/Alaska/20MB02534/2020 (H13N6)	MZ015703.1	74.2	QUE37851.1	83.4	91
A/great black-headed gull/Atyrau/743/2004 (H13N6)	GU982281.1	93.9	ADD92017.1	94.5	97.3
A/Laughing Gull/Delaware/294/2021 (H13N6)	OM966178.1	93.5	UNA44787.1	94.9	97
A/Mongolian gull/Mongolia/405/2007 (H13N6)	GQ907318.1	94.29	ACV86850.1	94.3	97.3
A/Kelp gull/Peru/M8/2019 (H13N6)	OL355043.1	74.5	UEE12803.1	83.6	91
A/silver gull/Tasmania/06-0349-105/2006 (H13N6)	OL369948.1	79.7	UEF69690.1	88	95.4
A/yellow-legged gull/Georgia/1/2011 (H13N6)	CY185497.1	95.22	AHZ39237.1	94.7	97
A/yellow-legged gull/Republic of Georgia/3/2012 (H13N6)	CY185611.1	93.9	AHZ39451.1	95.2	97.5
**Anatidae**					
A/duck/Siberia/272PF/1998 (H13N6)	AB285094.1	91.69	BAF38383.1	94.3	97
A/duck/Hokkaido/W189/2006 (H13N6)	LC339627.1	91.2	BBB38800.1	94.5	97
A/hooded merganser/New Brunswick/03750/2009 (H13N6)	CY125309.1	76.7	AFP21003.1	86.4	93.1
A/mallard/Dalian/DZ-137/2013 (H13N6)	KJ907711.1	89.4	AID48136.1	94.2	97.3
A/shelduck/Ukraine/KT-9-2-11/2016 (H13N6)	MW132954.1	74.4	QOL24253.1	82.7	90.6
A/whistling swan/Shimane/1343/1981 (H13N6)	LC336770.1	88.81	BBB38383.1	88.2	91.9
**Other wild birds**					
A/Eurasian curlew/Liaoning/ZH-186/2014 (H13N6)	KR010435.1	92.7	AKD00247.1	94.7	97.5
A/Red Knot/Delaware/224/2021 (H13N6)	OM965834.1	93.4	UNA44627.1	94.9	97.3
A/Red Knot/Delaware Bay/605/2020 (H13N6)	MW874987.1	76.9	QTO33855.1	85.9	93.3
A/ruddy turnstone/New Jersey/AI01-1407/2001 (H13N6)	MH500865.1	76.1	AWV92012.1	86.6	93.1
A/ruddy turnstone/New Jersey/UGAI14-1436/2014 (H13N6)	MH502664.1	77	AWV94705.1	86.2	93.5
A/shorebird/Delaware/224/1997 (H13N6)	KF612952.1	74.1	AGW82834.1	85	91
**H13N6 Reference isolate**					
A/gull/Maryland/704/1977 (H13N6)	CY130086.1	87	AGB51312.1	92.6	96.3

**Table 3:** List of amino acid mutations of SKH13N6 to the closest reference, obtained using FluSurver.

SKH13N6 Accession ID	Gene Segment	Sequence Length (aa)	Best Reference Hit	Accession ID	Identity (%)	Number of Mutations	List of Mutations
WHT33238.1	PB2	759	A/Mallard/Astrakhan/263/1982 (H14N5)	EPI_ISL_132864	97.628	18	E6D, D60E, T106A, S107N, I147V, I185V, L374I, M444V, N456S, V478I, M483T, V511I, S629N, R630K, L636M, V667I, V686I, I743R
WHT33236.1	PB1	757	A/Shearwater/Australia/2576/1979 (H15N9)	EPI_ISL_132866	98.151	14	K54T, T57I, V113I, M171L, E172D, E178G, E180D, Q210H, N213S, N328K, K388E, E390D, K391N, I667V
WHT33234.1	PA	716	A/Duck/Guangdong/E1/2012 (H10N8)	EPI_ISL_123953	98.883	8	L105F, R158K, M211V, R230T, I323V, I348L, N350S, E352D
WHT33227.1	HA	566	A/Gull/Maryland/704/1977 (H13N6)	EPI_ISL_132863	92.58	42	A7T, T8I, I12M, S13N, C15Y, H17Q, S87N, A102G, R127K, T138N, T147A, N154D, T155R, N158S, I168V, V199I, D202E, I250L, F264L, R285H, R290K, I409F, L423F, T432P, W435C, L442F, A456V, K459R, R466G, D471N, D475E, L483F, L484F, Y500S, D501N, E504D, D517N, I519V, S539T, L546F, S557N, N559S
WHT33229.1	NP	498	A/Gull/Maryland/704/1977 (H13N6)	EPI_ISL_132863	97.189	14	D18E, N23T, K77R, T85A, D101E, A129S, N375S, T396N, V406I, T433A, N450S, S451A, S482N, N483K
WHT33228.1	NA	469	A/Gull/Maryland/704/1977 (H13N6)	EPI_ISL_132863	92.964	35	V39A, G40C, T42A, V45S, N46P, T47S, P48S, V50G, S55del, Q66del, I76V, H77Q, N78I, R83K, E94A, E112N, R144Q, A173V, I175V, I189R, V214I, K236N, V242I, A253T, E269D, E287G, T315S, I323V, N346G, N387S, G391S, I393T, T394A, I398V, R433K
WHT33230.1	M1	252	A/Gull/Maryland/704/1977 (H13N6)	EPI_ISL_132863	99.206	2	V63I, N231D
WHT33231.1	M2	97	A/Hubei/1/2010 (H5N1)	AEO89185	93.814	6	T11I, K13S, R18K, V28I, L55I, G58E
WHT33232.1	NS1	230	A/herring gull/New_Jersey/780/1986 (H16N3)	EPI_ISL_132863	88.261	27	E26D, I36L, I48S, A60S, D70G, N80T, P87S, V90I, P95T, R118K, L124I, N127D, V129T, A137I, D139N, I145V, S152N, I180V, V194I, T197N, S205I, A215T, K217E, E219K, R220Q, M222L, G224R
WHT33233.1	NEP	121	A/Gull/Maryland/704/1977(H13N6)	EPI_ISL_132863	94.215	7	V14A, L40I, A48S, L57S, G63R, K64N, I76M

PB2, polymerase basic 2; PB1, polymerase basic 1; PA, polymerase acidic; HA, hemagglutinin; NP, nucleoprotein; NA, neuraminidase; M, matrix; NS, nonstructural.

**Table 4:** Identification of virulence- and drug-resistance-related amino acid substitutions in SKH13N6.

Predicted Activity	Gene Segment	Position	Amino Acid Mutation	SKH13N6 Residue	Target	Reference
Receptor binding						
	HA ^1^	193	K193T	T	↑ binding to human-type receptor (α2-6)	[26]
		228	G228S	S	↑ binding to human-type receptor (α2-6)	[27]
Drug resistance						
	NA ^2^	117	I117T	T	Oseltamivir and zanavir	[28]
Virulence						
	NS1	42	P42S	S	↑ virulence in mice	[29,30]
		149	V149A	A	↑ virulence in chickens	[31]
	NP	105	M105V	V	↑ virulence in chickens	[32]
		184	A184K	K	↑ virulence in chickens	[33]
	PA	37	S37A	A	↑ polymerase activity in mammalian cells	[34]
		190	P190S	S	↓ virulence in mice	[35]
		383	N383D	D	↑ polymerase activity in avian and human cells	[36]
		409	N409S	S	↑ polymerase activity and replication in mammalian cells	[34]
		550	I550L	L	↑ polymerase activity and virulence in mice	[37]
	M1	30	N30D	D	↑ virulence in mice	[38]
		215	T215A	A	↑ virulence in mice	
	PB2	89	L89V	V	↑ polymerase activity in mammalian cell line and virulence in mice	[39]
		292	I292V	V	↑ pathogenicity and replication in mammalian host	[40]
		309	G309D	D	↑ polymerase activity in mammalian cell line and virulence in mice	[39]
		339	T339K	K	↑ polymerase activity in mammalian cell line and virulence in mice	
		477	R477G	G	↑ polymerase activity in mammalian cell line and virulence in mice	
		495	I495V	V	↑ polymerase activity in mammalian cell line and virulence in mice	
		504	I504V	V	↑ polymerase activity in mice	[37]
		676	A676T	T	↑ polymerase activity in mammalian cell line and virulence in mice	[39]
Virulence	PB1	3	D3V	V	↑ polymerase activity and replication in mammalian and avian cells	[41]
		436	H436Y	Y	↑ polymerase activity in mammalian cell line, virulence in ducks, ferrets and mice	[42]
		622	D622G	G	↑ polymerase activity and virulence in mice	[43]

^1^ H3 numbering; ^2^ N2 numbering; PB2, polymerase basic 2; PB1, polymerase basic 1; PA, polymerase acidic; HA, hemagglutinin; NP, nucleoprotein; NA, neuraminidase; M, matrix; NS, nonstructural; ↑, increased; ↓, decreased.

**Table 5:** Percent identity and similarity of SKH13N6 NA gene to other known H13N6 isolates.

Isolate ID	Nucleotide		Amino acid		
	Accession	Identity (%)	Accession	Identity (%)	Similarity (%)
**Gulls**					
A/Armenian gull/Republic of Georgia/1/2012 (H13N6)	CY185357.1	96.5	AHZ39561.1	97.9	98.7
A/black headed gull/Mongolia/1766/2006 (H13N6)	GQ907304.1	94.96	ACV86833.1	95.9	97.4
A/black-headed gull/Republic of Georgia/7/2011 (H13N6)	CY185491.1	97.34	AHZ39228.1	97.9	98.7
A/black-headed gull/Netherlands/26/2014 (H13N6)	KX977752.1	95.5	APC30543.1	98.1	98.7
A/Glaucous-Winged Gull/Alaska/20MB02831/2020 (H13N6)	MZ015635.1	96.7	QUE37740.1	98.5	98.9
A/Glaucous-Winged Gull/Alaska/20MB02534/2020 (H13N6)	MZ015698.1	98.33	QUE37844.1	98.7	98.9
A/great black-headed gull/Atyrau/743/2004 (H13N6)	GU982285.1	93.4	ADD92021.1	96.2	97.4
A/Laughing Gull/Delaware/294/2021 (H13N6)	OM966171.1	77.2	UNA44776.1	86.2	93.2
A/Mongolian gull/Mongolia/405/2007 (H13N6)	GQ907320.1	93.1	ACV86853.1	95.7	97.9
A/Kelp gull/Peru/M8/2019 (H13N6)	OL355045.1	76.4	UEE12805.1	85.3	92.8
A/silver gull/Tasmania/06-0349-105/2006 (H13N6)	OL369950.1	87.4	UEF69692.1	90.4	93.6
A/yellow-legged gull/Georgia/1/2011 (H13N6)	CY185499.1	97.62	AHZ39240.1	97.7	98.5
A/yellow-legged gull/Republic of Georgia/3/2012 (H13N6)	CY185611.1	96.5	AHZ39454.1	97.4	98.5
**Anatidae**					
A/duck/Siberia/272PF/1998 (H13N6)	AB285096.1	92.1	BAF38385.1	94.5	97
A/duck/Hokkaido/W189/2006 (H13N6)	LC339629.1	91.2	BBB38802.1	93.6	96.4
A/hooded merganser/New Brunswick/03750/2009 (H13N6)	CY125311.1	76.6	AFP21006.1	86.4	93.2
A/mallard/Dalian/DZ-137/2013 (H13N6)	KJ907713.1	90.67	AID48138.1	92.1	95.7
A/shelduck/Ukraine/KT-9-2-11/2016 (H13N6)	MW132952.1	94.9	QOL24250.1	97.4	98.7
**Other wild birds**					
A/Eurasian curlew/Liaoning/ZH-186/2014 (H13N6)	KR010437.1	97.3	AKD00249.1	98.1	98.5
A/Red Knot/Delaware/224/2021 (H13N6)	OM965827.1	77.4	UNA44616.1	86.2	93.2
A/Red Knot/Delaware Bay/605/2020 (H13N6)	MW874986.1	76.6	QTO33854.1	86	93.2
A/ruddy turnstone/New Jersey/AI01-1407/2001 (H13N6)	MH500867.1	88.7	AWV92015.1	92.1	95.7
A/ruddy turnstone/New Jersey/UGAI14-1436/2014 (H13N6)	MH502666.1	76.9	AWV94708.1	85.7	93
A/shorebird/Delaware/224/1997 (H13N6)	CY015148.1	89	ABI85203.1	92.6	96
**H13N6 Reference isolate**					
A/gull/Maryland/704/1977 (H13N6)	CY130088.1	87.7	AGB51315.1	92.6	95.3

**Table 6:** Estimated dates of the most recent common ancestor (TMRCA) of SKH13N6 by gene segments.

Gene Segment	TMRCA (95% HPD Interval)			
	Mean	95% Lower HPD	95% Higher HPD	Posterior Probability
PB2	2013.4985	2012.3697	2014.6044	0.8874
PB1	2015.4666	2014.7576	2015.9193	1.0000
PA	2018.6772	2016.9407	2020.1785	1.0000
HA	2009.5347	2008.5285	2010.4524	1.0000
NP	2015.8315	2013.7787	2017.6613	1.0000
NA	2010.5671	2009.8727	2010.9990	0.9963
M	2015.591	2012.0967	2018.3715	0.5059
NS	2015.4673	2014.0530	2016.0000	1.0000

HPD, highest posterior density; PB2, polymerase basic 2; PB1, polymerase basic 1; PA, polymerase acidic; HA, hemagglutinin; NP, nucleoprotein; NA, neuraminidase; M, matrix; NS, nonstructural.

## Data Availability

All data are contained within the article and/or in the Supplementary Data.

## Supplementary Materials

The following supporting information can be downloaded at https://www.mdpi.com/article/10.3390/v16020285/s1: Table S1: Lists of primers used for the amplification of the eight vRNA segments of Influenza A virus; Table S2–S9: List of isolates used in this study for PB2, PB1, PA, HA, NP, NA, M, and NS gene analysis; Table S10: New amino acid variants found on the gene sequences of SKH13N6 compared to other H13N6 isolates in the Influenza Virus Database and GenBank; Table S11: New amino acid variants found on the internal gene sequences of SKH13N6 compared to the H13N6 reference virus (MDH13N6); Figure S1–S8: Phylogenetic tree of PB2, PB1, PA, HA, NP, NA, M, and NS gene segments by host. The maximum clade credibility (MCC) tree highlights the host trait of each gene segments. The cluster of SKH13N6 isolate is highlighted and the isolate is colored in red. Node bars indicate 95% highest posterior density.
