# Supplementary material for: Genetic Characterization and Phylogeographic Analysis of the First H13N6 Avian Influenza Virus Isolated from Vega Gull in South Korea
Source: Viruses. 2024 Feb 12;16(2):285. doi: 10.3390/v16020285 (PMC10891532; doi:10.3390/v16020285)
Supplement: Supplementary file 1 [file viruses-16-00285-s001.zip › Figure S7.pdf]

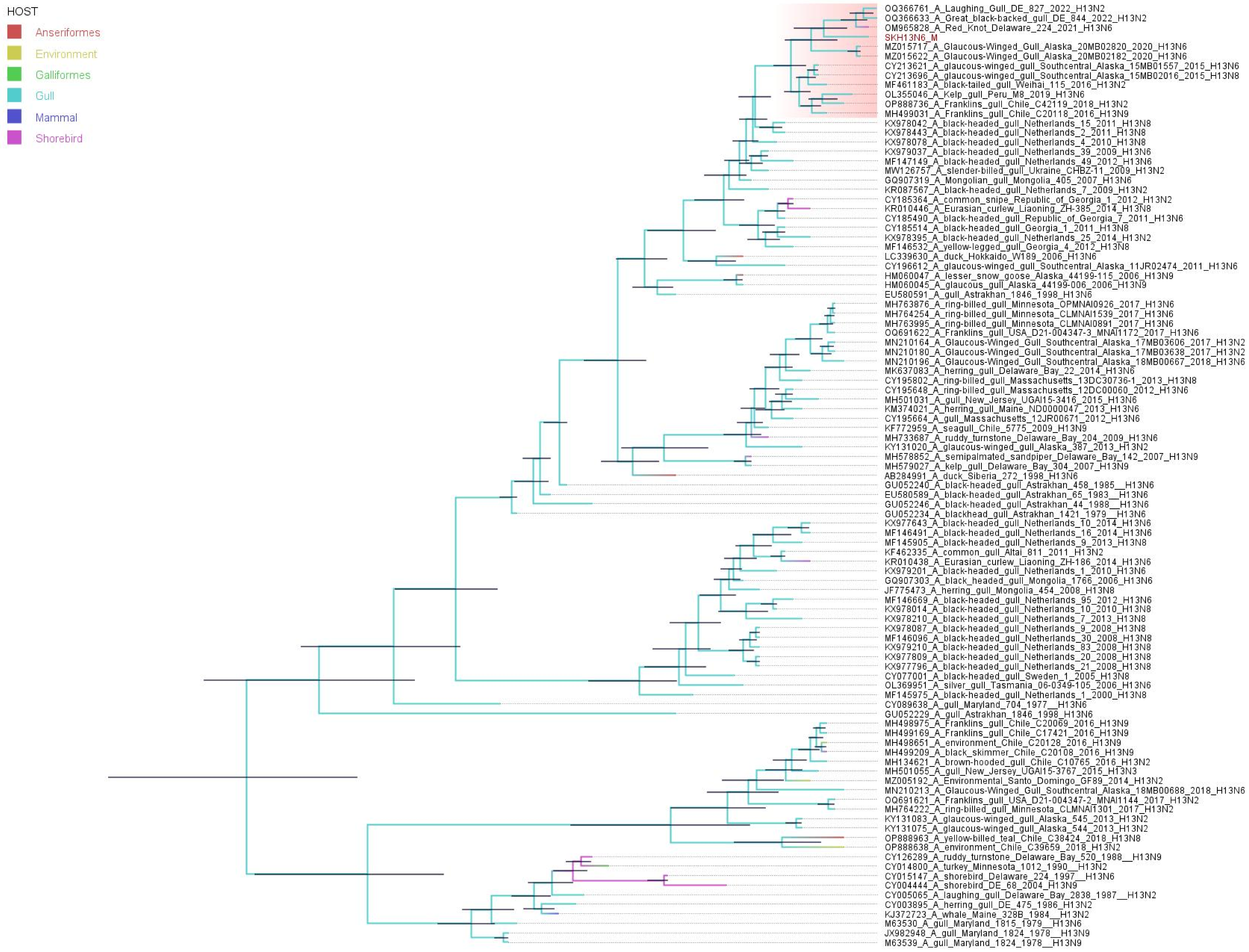

Figure S7. Phylogenetic tree of M gene segment by host. Maximum Clade Credibility (MCC) tree highlights the host trait of H13Nx isolates M gene segment (n=99). The cluster of SKH13N6 isolate is highlighted and the isolate is colored in red. Node bars indicate 95% Highest Posterior Density.
