# Supplementary material for: Genetic Characterization and Phylogeographic Analysis of the First H13N6 Avian Influenza Virus Isolated from Vega Gull in South Korea
Source: Viruses. 2024 Feb 12;16(2):285. doi: 10.3390/v16020285 (PMC10891532; doi:10.3390/v16020285)
Supplement: Supplementary file 1 [file viruses-16-00285-s001.zip › Table S1 Primers.pdf]

Table S1. Lists of primers used for the amplification of the eight vRNA segments of Influenza A virus

Name	Purpose	Sequence (5'-3')[a]	Target segment	Size (bp)	Reference
Uni12	cDNA synthesis	AGCAAAAGCAGG	All		Hoffmann, E.; Stech, J.; Guan, Y.; Webster, R.G.; Perez, D.R. Universal Primer Set for the Full-Length Amplification of All Influenza A Viruses. Arch. Virol. 2001, 146, 2275–2289.
Bm-NP-1<br>Bm-NP-1565R	PCR	AGCAAAAGCAGGGTA<br>AGTAGAAACAAGGGTATTTTT	NP	1565	
Bm-M-1F<br>Bm-M-1027R	PCR	AGCAAAAGCAGGTAG<br>AGTAGAAACAAGGTAGTTTTT	MA	1027	
Bm-NS-1F<br>Bm-NS-890R	PCR	AGCAAAAGCAGGGTG<br>AGTAGAAACAAGGGTGTTTT	NS	890	
Bm-PA-1<br>PA-1498R<br>PA-747F<br>Bm-PA-2233R	PCR	TATTCGTCTCAGGGAGCGAAAGCAGGTAC<br>TNGTYCTRCAYTTGCTTATCAT<br>CATTGAGGGCAAGCTTTC<br>ATATCGTCTCGTATTAGTAGAAACAAGGTACTT	PA	2233	Li, O.T.W.; Barr, I.; Leung, C.Y.H.; Chen, H.; Guan, Y.; Peiris, J.S.M.; Poon, L.L.M. Reliable Universal RT-PCR Assays for Studying Influenza Polymerase Subunit Gene Sequences from All 16 Haemagglutinin Subtypes. J. Virol. Methods 2007, 142, 218–222.
Bm-PB1-1<br>PB1-1262R<br>PB1-1124F<br>Bm-PB1-2341R	PCR	TATTCGTCTCAGGGAGCGAAAGCAGGCA<br>TTRAACATGCCCATCATCAT<br>ARATACCNGCAGARATGCT<br>ATATCGTCTCGTATTAGTAGAAACAAGGCA	PB1	2341	
Bm-PB2-1<br>PB2-1250R<br>PB2-1105F<br>Bm-PB2-2341R	PCR	TATTGGTCTCAGGGAGCGAAAGCAGGTC<br>TCYTCYTGTGARAAYACCAT<br>TAYGARGARTTCACAATGGT<br>ATATGGTCTCGTATTAGTAGAAACAAGGTCG	PB2	2341	
HA-1<br>HA-1778R	PCR	AGCAAAAGCAGGGG<br>AGTAGAAACAAGGGTGTTTT	HA	1778	Chan, C.H.; Lin, K.L.; Chan, Y.; Wang, Y.L.; Chi, Y.T.; Tu, H.L.; Shieh, H.K.; Liu, W.T. Amplification of the Entire Genome of Influenza A Virus H1N1 and H3N2 Subtypes by Reverse-Transcription Polymerase Chain Reaction. J. Virol. Methods 2006, 136, 38–43.
AIV-NA-BO-F13<br>AIV-NA-BO-FII1<br>AIV-NA-BO-R	PCR	GGGAGCAAAAGCAGGAGTRAARATGAA<br>GGGAGCAAAAGCAGGKKSRMDATGAATC<br>GGGAGTAGAAACAAGGDBNTTTTTB	NA	1413	Orozovic, G.; Latorre-Margalef, N.; Wahlgren, J.; Muradrasoli, S.; Olsen, B. Degenerate Primers for PCR Amplification and Sequencing of the Avian Influenza A Neuraminidase Gene. J. Virol. Methods 2010, 170, 94–98.

[a]Codes for mixed bases position: R= A/G, Y=C/T
