# Supplementary material for: Genetic Characterization and Phylogeographic Analysis of the First H13N6 Avian Influenza Virus Isolated from Vega Gull in South Korea
Source: Viruses. 2024 Feb 12;16(2):285. doi: 10.3390/v16020285 (PMC10891532; doi:10.3390/v16020285)
Supplement: Supplementary file 1 [file viruses-16-00285-s001.zip › Table S10.pdf]

Table S10. New amino acid variants found on the gene sequences of SKH13N6 compared to other H13N6 isolates in Influenza Virus Database and GenBank

Genes[1]	Position	Database consensus	SKH13N6
Accession			WHT33227
	13	G/S/R	N
	199	V	I
	409	I	F
	423	L	F
	432	T	P
	435	W	C
	442	L	F
**HA**	456	A	V
(n258)	466	R/K/Q	G
	475	D	E
	483	LL	FF
	500	Y	S
	504	E/K	D
	539	S	T
	546	L/P	F
Accession			WHT33228
**NA**	253	A	T
(n224)	391	G	S
Accession			WHT33229
**NP**	396	T	N
(n293)			
Accession			WHT33232
**NS1**	205	S	I
(n295)			
Accession			WHT33234
**PA**	211	M	V
(n275)	230	R	T
Accession			WHT33235
**PA-X**	230	E	Q
(n250)			
Accession			WHT33238
	6	E	D
**PB2**	629	S	N
(n282)	743	I	R

[1]Number of analyzed sequences is listed below the gene name
