# Supplementary material for: Genetic Characterization and Phylogeographic Analysis of the First H13N6 Avian Influenza Virus Isolated from Vega Gull in South Korea
Source: Viruses. 2024 Feb 12;16(2):285. doi: 10.3390/v16020285 (PMC10891532; doi:10.3390/v16020285)
Supplement: Supplementary file 1 [file viruses-16-00285-s001.zip › Table S11.pdf]

Table S11. New amino acid variants found on the internal gene sequences of SKH13N6 compared to H13N6 reference virus (MDH13N6).

Genes	Position	H13N6 Reference A/gull/Maryland/704/1977	SKH13N6	Pairwise Identity (%)	Pairwise Similarity (%)
Accession		AGB51313.1	WHT33230		
M1	63	V	I	99.2	100
	231	N	D		
Accession		AGB51314.1	WHT33231		
M2	13	N	S	91.8	94.8
	18	R	K		
	47	L	F		
	50	F	C		
	55	F	I		
	58	G	E		
	65	M	T		
	82	S	N		
Accession		AGB51316.1	WHT33229		
NP	18	D	E	97.2	99
	23	N	T		
	77	K	R		
	85	T	A		
	101	D	E		
	129	A	S		
	375	N	S		
	396	T	N		
	406	V	I		
	433	T	A		
	450	NS	SA		
	482	SN	NK		
Accession		AGB51317.1	WHT33232		
NS1	26	E	D	87.8	95.2
	36	I	L		
	48	I	S		
	60	A	S		
	63	L	Q		
	70	D	G		
	80	N	T		
	90	V	I		
	95	P	T		
	118	R	K		
	124	L	I		
	127	N	D		
	129	V	T		
	137	A	I		
	139	D	N		
	145	I	V		
	152	S	N		
	180	I	V		
	194	V	I		
	197	T	N		
	205	S	I		
	215	A	T		
	217	K	E		
	219	ER	KQ		
	222	M	L		
	224	G	R		
	226	V	I		
Accession		AGB51318.1	WHT33233		
NEP	14	V	A	94.2	96.7
	40	L	I		
	48	A	S		
	57	L	S		
	63	GK	RN		
	76	I	M		
Accession		AGB51319.1	WHT33234		
PA	101	E	D	98.5	99.6
	211	M	V		
	230	R	T		
	272	E	D		
	328	R	K		
	342	Q	L		
	348	I	L		
	350	N	S		
	391	K	R		
	400	Q	S		
	407	V	I		
Accession		AGB51320.1	WHT33236		
PB1	54	K	T	97.8	99.2
	57	T	I		
	113	V	I		
	171	ME	LD		
	178	E	G		
	180	E	D		
	210	Q	H		
	213	NRR	SKK		
	328	N	K		
	375	N	S		
	388	K	E		
	390	EK	DN		
	667	I	V		
Accession		AGB51319.1	WHT33238		
PB2	6	E	D	96.7	99.3
	60	D	E		
	106	TS	AN		
	147	I	V		
	180	D	E		
	185	I	V		
	292	I	V		
	338	I	V		
	374	L	I		
	456	N	S		
	470	NA	ST		
	483	M	T		
	511	V	I		
	559	MI	TV		
	567	N	D		
	629	SR	NK		
	636	L	M		
	667	V	I		
	674	S	A		
	686	V	I		
	743	I	R		
