# Supplementary material for: Genetic Characterization and Phylogeographic Analysis of the First H13N6 Avian Influenza Virus Isolated from Vega Gull in South Korea
Source: Viruses. 2024 Feb 12;16(2):285. doi: 10.3390/v16020285 (PMC10891532; doi:10.3390/v16020285)
Supplement: Supplementary file 1 [file viruses-16-00285-s001.zip › Table S2 PB2 isolates.pdf]

**Table S2.** List of isolates used in this study for PB2 gene analysis

| ISOLATE_ID | LOCATION | HOST |
|---|---|---|
| ABB87344_A_shorebird_DE_68_2004_H13N9 | North America | Shorebird |
| ABI84576_A_gull_Maryland_704_1977_H13N6 | North America | Gull |
| ACT84623_A_laughing_gull_New_Jersey_Sg-00568_2008_H13N9 | North America | Gull |
| ACU15645_A_shorebird_Delaware_221_2006_H13N9 | North America | Shorebird |
| ACV86839_A_black_headed_gull_Mongolia_1766_2006_H13N6 | Asia | Gull |
| ACV86849_A_Mongolian_gull_Mongolia_401_2007_H13N6 | Asia | Gull |
| ACV86859_A_Mongolian_gull_Mongolia_405_2007_H13N6 | Asia | Gull |
| ADM07074_A_glaucous-winged_gull_Southcentral_Alaska_9JR0747R0_2009_H13N6 | North America | Gull |
| ADM07096_A_glaucous-winged_gull_Southcentral_Alaska_9JR0781R0_2009_H13N6 | North America | Gull |
| ADQ26650_A_black-headed_gull_Sweden_1_2005_H13N8 | Europe | Gull |
| AEB71112_A_herring_gull_Mongolia_454_2008_H13N8 | Asia | Gull |
| AFL56746_A_mallard_Korea_SH38-45_2010_H13N2 | Asia | Anseriformes |
| AFP20880_A_ring-billed_gull_Quebec_02434-1_2009_H13N6 | North America | Gull |
| AFX86019_A_shorebird_Delaware_Bay_424_2007_H13N9 | North America | Shorebird |
| AFY06260_A_mallard-black_duck_hybrid_New_Brunswick_03736_2009_H13N6 | North America | Anseriformes |
| AGB98193_A_glaucous-winged_gull_Southeastern_Alaska_9JR0822R0_2009_H13N6 | North America | Gull |
| AGK42191_A_laughing_gull_New_Jersey_Sg-00568_2008_H13N9 | North America | Gull |
| AGL57558_A_ring-billed_gull_Georgia_AI00-2658_2000_H13N6 | Europe | Gull |
| AGL58712_A_laughing_gull_New_Jersey_Sg-00485_2008_H13N9 | North America | Gull |
| AGL60145_A_ruddy_turnstone_New_Jersey_AI09-294_2009_H13N6 | North America | Shorebird |
| AHA57185_A_seagull_Chile_5775_2009_H13N9 | South America | Gull |
| AHZ38838_A_black-headed_gull_Sweden_74342_2008_H13N8 | Europe | Gull |
| AHZ39236_A_black-headed_gull_Republic_of_Georgia_7_2011_H13N6 | Europe | Gull |
| AHZ39248_A_yellow-legged_gull_Georgia_1_2011_H13N6 | Europe | Gull |
| AHZ39295_A_black-headed_gull_Georgia_1_2011_H13N8 | Europe | Gull |
| AHZ39319_A_black-headed_gull_Georgia_3_2011_H13N8 | Europe | Gull |
| AHZ39355_A_black-headed_gull_Georgia_6_2011_H13N8 | Europe | Gull |
| AHZ39391_A_black-headed_gull_Republic_of_Georgia_2_2012_H13N6 | Europe | Gull |
| AHZ39462_A_yellow-legged_gull_Republic_of_Georgia_3_2012_H13N6 | Europe | Gull |
| AHZ39509_A_yellow-legged_gull_Georgia_2_2013_H13N8 | Europe | Gull |
| AHZ39569_A_Armenian_gull_Republic_of_Georgia_1_2012_H13N6 | Europe | Gull |
| AHZ39613_A_yellow-legged_gull_Republic_of_Georgia_6_2012_H13N6 | Europe | Gull |
| AID48133_A_mallard_Dalian_DZ-137_2013_H13N6 | Asia | Anseriformes |
| AIN76259_A_herring_gull_Maine_ND0000047_2013_H13N6 | North America | Gull |
| AKD00244_A_Eurasian_curlew_Liaoning_ZH-186_2014_H13N6 | Asia | Shorebird |
| ALZ46758_A_ring-billed_gull_Massachusetts_12DC00060_2012_H13N6 | North America | Gull |
| ALZ46782_A_gull_Massachusetts_12JR00671_2012_H13N6 | North America | Gull |
| ALZ46989_A_ring-billed_gull_Massachusetts_13DC30736-1_2013_H13N8 | North America | Gull |
| ALZ48201_A_glaucous-winged_gull_Southcentral_Alaska_11JR02474_2011_H13N6 | North America | Gull |
| APC30377_A_black-headed_gull_Netherlands_39_2009_H13N6 | Europe | Gull |
| APC30521_A_black-headed_gull_Netherlands_17_2014_H13N6 | Europe | Gull |
| APC30774_A_black-headed_gull_Netherlands_96_2008_H13N8 | Europe | Gull |
| APC30844_A_black-headed_gull_Netherlands_12_2010_H13N2 | Europe | Gull |
| APC30893_A_black-headed_gull_Netherlands_37_2009_H13N2 | Europe | Gull |
| APC30961_A_black-headed_gull_Netherlands_27_2014_H13N2 | Europe | Gull |
| APC30992_A_black-headed_gull_Netherlands_105_2012_H13N6 | Europe | Gull |
| APC31016_A_black-headed_gull_Netherlands_23_2014_H13N2 | Europe | Gull |
| APC31077_A_black-headed_gull_Netherlands_17_2009_H13N3 | Europe | Gull |
| APC31096_A_black-headed_gull_Netherlands_9_2008_H13N8 | Europe | Gull |
| APC31149_A_black-headed_gull_Netherlands_7_2013_H13N8 | Europe | Gull |
| APC31173_A_black-headed_gull_Netherlands_1_2014_H13N2 | Europe | Gull |
| APC31177_A_black-headed_gull_Netherlands_69_2012_H13N6 | Europe | Gull |
| APC31428_A_black-headed_gull_Netherlands_20_2008_H13N8 | Europe | Gull |
| APC31492_A_black-headed_gull_Netherlands_10_2014_H13N6 | Europe | Gull |
| APC31541_A_black-headed_gull_Netherlands_9_2009_H13N2 | Europe | Gull |
| APC31549_A_black-headed_gull_Netherlands_10_2013_H13N8 | Europe | Gull |
| APC31601_A_black-headed_gull_Netherlands_81_2012_H13N6 | Europe | Gull |
| APC31649_A_black-headed_gull_Netherlands_15_2011_H13N8 | Europe | Gull |
| APC31774_A_black-headed_gull_Netherlands_10_2007_H13N6 | Europe | Gull |
| APC31939_A_black-headed_gull_Netherlands_13_2010_H13N2 | Europe | Gull |
| APC31990_A_black-headed_gull_Netherlands_104_2012_H13N6 | Europe | Gull |
| APC32172_A_black-headed_gull_Netherlands_25_2008_H13N8 | Europe | Gull |
| APC32193_A_black-headed_gull_Netherlands_55_2008_H13N8 | Europe | Gull |
| APC32254_A_black-headed_gull_Netherlands_33_2009_H13N2 | Europe | Gull |
| APC32373_A_black-headed_gull_Netherlands_12_2014_H13N2 | Europe | Gull |
| APC32385_A_black-headed_gull_Netherlands_8_2010_H13N2 | Europe | Gull |
| APC32389_A_black-headed_gull_Netherlands_6_2010_H13N8 | Europe | Gull |
| APC32407_A_black-headed_gull_Netherlands_10_2010_H13N8 | Europe | Gull |
| APC32422_A_black-headed_gull_Netherlands_102_2008_H13N8 | Europe | Gull |
| APC32680_A_black-headed_gull_Netherlands_72_2012_H13N6 | Europe | Gull |
| APC33108_A_black-headed_gull_Netherlands_5_2014_H13N6 | Europe | Gull |
| APH09549_A_glaucous-winged_gull_Alaska_458_2013_H13N2 | North America | Gull |
| APH09573_A_glaucous-winged_gull_Alaska_544_2013_H13N2 | North America | Gull |
| APH09585_A_glaucous-winged_gull_Alaska_545_2013_H13N2 | North America | Gull |
| APH09681_A_glaucous-winged_gull_Alaska_695_2013_H13N2 | North America | Gull |
| APY16429_A_glaucous-winged_gull_Southcentral_Alaska_15MB01610_2015_H13N6 | North America | Gull |
| APY16569_A_glaucous-winged_gull_Southcentral_Alaska_15MB01557_2015_H13N6 | North America | Gull |
| APY16581_A_glaucous-winged_gull_Southcentral_Alaska_15MB01429_2015_H13N6 | North America | Gull |
| APY16593_A_glaucous-winged_gull_Southcentral_Alaska_15MB01632_2015_H13N6 | North America | Gull |
| ARX91538_A_black-headed_gull_Netherlands_38_2011_H13N8 | Europe | Gull |
| ARX91677_A_black-headed_gull_Netherlands_18_2012_H13N6 | Europe | Gull |
| ARX91698_A_black-headed_gull_Netherlands_84_2012_H13N6 | Europe | Gull |
| ARX91955_A_black-headed_gull_Netherlands_64_2008_H13N8 | Europe | Gull |

| | | |
|---|---|---|
| ARX92114_A_black-headed_gull_Netherlands_48_2012_H13N6 | Europe | Gull |
| ARX92210_A_black-headed_gull_Netherlands_8_2009_H13N2 | Europe | Gull |
| ARX92308_A_black-headed_gull_Netherlands_16_2014_H13N6 | Europe | Gull |
| ARX92327_A_black-headed_gull_Netherlands_13_2008_H13N8 | Europe | Gull |
| ARX92368_A_black-headed_gull_Netherlands_4_2012_H13N6 | Europe | Gull |
| ARX92535_A_black-headed_gull_Netherlands_2_2009_H13N2 | Europe | Gull |
| ARX92986_A_black-headed_gull_Netherlands_49_2008_H13N8 | Europe | Gull |
| ARX93456_A_black-headed_gull_Netherlands_39_2008_H13N8 | Europe | Gull |
| ARX93480_A_yellow-legged_gull_Georgia_1_2011_H13N8 | Europe | Gull |
| ARX93541_A_black-headed_gull_Netherlands_3_2011_H13N8 | Europe | Gull |
| ARX93960_A_black-headed_gull_Netherlands_36_2012_H13N6 | Europe | Gull |
| ARX93970_A_black-headed_gull_Netherlands_6_2013_H13N8 | Europe | Gull |
| ARX94095_A_black-headed_gull_Netherlands_63_2012_H13N6 | Europe | Gull |
| ARX94303_A_yellow-legged_gull_Georgia_4_2012_H13N8 | Europe | Gull |
| ARX94327_A_black-headed_gull_Netherlands_19_2012_H13N6 | Europe | Gull |
| ARX94412_A_black-headed_gull_Netherlands_39_2011_H13N8 | Europe | Gull |
| ARX94421_A_black-headed_gull_Netherlands_1_2000_H13N8 | Europe | Gull |
| ARX94428_A_black-headed_gull_Netherlands_6_2012_H13N6 | Europe | Gull |
| ARX94567_A_black-headed_gull_Netherlands_36_2009_H13N2 | Europe | Gull |
| ARX94713_A_black-headed_gull_Netherlands_21_2012_H13N6 | Europe | Gull |
| ASN77228_A_herring_gull_Massachusetts_A00080257_2006_H13N2 | North America | Gull |
| ASO97740_A_black-tailed_gull_Weihai_115_2016_H13N2 | Asia | Gull |
| ASO97750_A_black-tailed_gull_Weihai_17_2016_H13N8 | Asia | Gull |
| AVO00179_A_laughing_gull_New_Jersey_UGAI17-2839_2017_H13N6 | North America | Gull |
| AVO00215_A_laughing_gull_New_Jersey_UGAI17-2850_2017_H13N6 | North America | Gull |
| AWF93161_A_environment_Chile_C7039_2016_H13N2 | South America | Environment |
| AWF93218_A_environment_Chile_C7125_2016_H13N2 | South America | Environment |
| AWF93299_A_brown-hooded_gull_Chile_C10256_2016_H13N2 | South America | Gull |
| AWF93597_A_environment_Chile_C7144_2016_H13N2 | South America | Environment |
| AWV92023_A_ruddy_turnstone_New_Jersey_AI01-1407_2001_H13N6 | North America | Shorebird |
| AWV92269_A_gull_New_Jersey_UGAI15-3416_2015_H13N6 | North America | Gull |
| AWV92305_A_gull_New_Jersey_UGAI15-3767_2015_H13N3 | North America | Gull |
| AWV93209_A_ruddy_turnstone_New_Jersey_AI12-1737_2012_H13N6 | North America | Shorebird |
| AWW20777_A_environment_Valparaiso_P11_2017_H13N9 | South America | Environment |
| AXB35135_A_environment_New_Jersey_AI00-2413_2000_H13N6 | North America | Environment |
| AXB35147_A_herring_gull_New_Jersey_AI07-418_2007_H13N9 | North America | Gull |
| AXE75375_A_environment_Chile_C20076_2016_H13N9 | South America | Environment |
| AXF48992_A_American_oystercatcher_Chile_C20102_2016_H13N9 | South America | Shorebird |
| AXF49019_A_Franklins_gull_Chile_C17421_2016_H13N9 | South America | Gull |
| AXF49110_A_Franklins_gull_Chile_C20110_2016_H13N9 | South America | Gull |
| AXF49119_A_Franklins_gull_Chile_C20118_2016_H13N9 | South America | Gull |
| AXF49140_A_Franklins_gull_Chile_C20373_2016_H13N9 | South America | Gull |
| AXF49155_A_black_skimmer_Chile_C20057_2016_H13N8 | South America | Shorebird |
| AXF49168_A_black_skimmer_Chile_C20077_2016_H13N9 | South America | Shorebird |
| AXF49174_A_black_skimmer_Chile_C20084_2016_H13N9 | South America | Shorebird |
| AXF49194_A_black_skimmer_Chile_C20100_2016_H13N9 | South America | Shorebird |
| AXF49327_A_environment_Chile_C20059_2016_H13N9 | South America | Environment |
| AXF49355_A_environment_Chile_C20126_2016_H13N9 | South America | Environment |
| AXF49409_A_environment_Chile_C20145_2016_H13N9 | South America | Environment |
| AXF49434_A_environment_Chile_C20148_2016_H13N9 | South America | Environment |
| AXF49468_A_environment_Chile_C20173_2016_H13N9 | South America | Environment |
| AXF49557_A_environment_Chile_C20182_2016_H13N9 | South America | Environment |
| AXF49591_A_kelp_gull_Chile_C20137_2016_H13N9 | South America | Gull |
| AXF49609_A_kelp_gull_Chile_C27733_2017_H13N8 | South America | Gull |
| AXF49632_A_kelp_gull_Chile_C8595_2016_H13N2 | South America | Gull |
| AXF49733_A_whimbrel_Chile_C20106_2016_H13N9 | South America | Shorebird |
| AXF49754_A_whimbrel_Chile_C20147_2016_H13N9 | South America | Shorebird |
| AXK58617_A_semipalmated_sandpiper_Delaware_Bay_142_2007_H13N9 | North America | Shorebird |
| AXO65670_A_ring-billed_gull_Minnesota_CLMNAI1199_2017_H13N2 | North America | Gull |
| AXO65682_A_ring-billed_gull_Minnesota_CLMNAI0790_2017_H13N8 | North America | Gull |
| AXO65766_A_ring-billed_gull_Minnesota_CLMNAI0833_2017_H13N8 | North America | Gull |
| AXO65778_A_ring-billed_gull_Minnesota_CLMNAI0939_2017_H13N6 | North America | Gull |
| AXO65814_A_ring-billed_gull_Minnesota_CLMNAI0968_2017_H13N8 | North America | Gull |
| AXO65870_A_ring-billed_gull_Minnesota_CLMNAI1301_2017_H13N2 | North America | Gull |
| AXO65894_A_ring-billed_gull_Minnesota_CLMNAI1501_2017_H13N2 | North America | Gull |
| AXO65906_A_ring-billed_gull_Minnesota_CLMNAI1529_2017_H13N6 | North America | Gull |
| AZR38848_A_laughing_gull_Delaware_Bay_451_2018_H13N6 | North America | Gull |
| AZV03297_A_laughing_gull_Delaware_Bay_433_2018_H13N6 | North America | Gull |
| AZZ86188_A_kelp_gull_Delaware_Bay_441_2018_H13N6 | North America | Gull |
| BAF37817_A_duck_Siberia_272_1998_H13N6 | Asia | Anseriformes |
| BBB38796_A_duck_Hokkaido_W189_2006_H13N6 | Asia | Anseriformes |
| CCH50634_A_common_gull_Norway_10_1313_2009_H13N2 | Europe | Gull |
| QBL97181_A_red_knot_Delaware_Bay_489_2010_H13N6 | North America | Shorebird |
| QBM13374_A_laughing_gull_Delaware_Bay_350_2014_H13N6 | North America | Gull |
| QBM14655_A_herring_gull_Delaware_Bay_22_2014_H13N6 | North America | Gull |
| QBM14717_A_laughing_gull_Delaware_Bay_39_2014_H13N6 | North America | Gull |
| QBM15097_A_laughing_gull_Delaware_Bay_328_2014_H13N6 | North America | Gull |
| QDP69640_A_Glaucous_Gull_Northern_Alaska_18AR00289_2018_H13N6 | North America | Gull |
| QDP69676_A_Glaucous-Winged_Gull_Southcentral_Alaska_17MB03606_2017_H13N2 | North America | Gull |
| QDP69688_A_Glaucous-Winged_Gull_Southcentral_Alaska_17MB03625_2017_H13N2 | North America | Gull |
| QDP69700_A_Glaucous-Winged_Gull_Southcentral_Alaska_17MB03638_2017_H13N2 | North America | Gull |
| QDP69724_A_Glaucous-Winged_Gull_Southcentral_Alaska_18MB00667_2018_H13N6 | North America | Gull |
| QDP69762_A_Glaucous-Winged_Gull_Southcentral_Alaska_18MB00689_2018_H13N6 | North America | Gull |
| QDP69834_A_Herring_Gull_Massachusetts_A00648685_2008_H13N2 | North America | Gull |
| QDP69846_A_Herring_Gull_Massachusetts_A00648696_2008_H13N2 | North America | Gull |
| QGR25239_A_Red_Knot_Delaware_Bay_3_2019_H13N6 | North America | Shorebird |
| QOL20611_A_slender-billed_gull_Ukraine_CHBZ-11_2009_H13N2 | Europe | Gull |

| | | |
|---|---|---|
| QOL24246_A_shelduck_Ukraine_KT-9-2-11_2016_H13N6 | Europe | Anseriformes |
| QTO29226_A_glaucous-winged_gull_Alaska_AK18-288_2018_H13N6 | North America | Gull |
| QTO33865_A_Red_Knot_Delaware_Bay_605_2020_H13N6 | North America | Shorebird |
| QUD20217_A_Environmental_Santo_Domingo_GF89_2014_H13N2 | South America | Environment |
| QUE37728_A_Glaucous-Winged_Gull_Alaska_20MB02182_2020_H13N6 | North America | Gull |
| QUE37801_A_Glaucous-Winged_Gull_Alaska_20MB02498_2020_H13N6 | North America | Gull |
| SKH13N6_PB2 | Asia | Gull |
| UEE12798_A_Kelp_gull_Peru_M8_2019_H13N6 | South America | Gull |
| UEF69685_A_silver_gull_Tasmania_06-0349-105_2006_H13N6 | Ocenia | Gull |
| UNA44626_A_Red_Knot_Delaware_224_2021_H13N6 | North America | Shorebird |
| UNA44786_A_Laughing_Gull_Delaware_294_2021_H13N6 | North America | Gull |
| UZU88878_A_kelp_gull_Chile_C25097_2017_H13N9 | South America | Gull |
| UZU88948_A_kelp_gull_Chile_C39002_2018_H13N2 | South America | Gull |
| UZU88995_A_white-backed_stilt_Chile_C21965_2017_H13N9 | South America | Shorebird |
| UZU89019_A_environment_Chile_C39659_2018_H13N2 | South America | Environment |
| UZU89086_A_kelpgull_Chile_C24014_2017_H13N9 | South America | Gull |
| UZU89143_A_kelp_gull_Chile_C41225_2018_H13N6 | South America | Gull |
| UZU89213_A_Franklins_gull_Chile_C38092_2018_H13N2 | South America | Gull |
| UZU89267_A_environment_Chile_C39624_2018_H13N2 | South America | Environment |
| UZU89315_A_southern_lapwing_Chile_C36480_2018_H13N8 | South America | Shorebird |
| UZU89395_A_yellow-billed_teal_Chile_C37436_2018_H13N2 | South America | Anseriformes |
| UZU89421_A_Franklins_gull_Chile_C33636_2017_H13N2 | South America | Gull |
| UZU89502_A_yellow-billed_teal_Chile_C38424_2018_H13N8 | South America | Anseriformes |
| WCJ14712_A_Great_black-backed_gull_DE_844_2022_H13N2 | North America | Gull |
| WCJ14792_A_Laughing_Gull_DE_801_2022_H13N2 | North America | Gull |
| WCJ14985_A_Iceland_Gull_DE_819_2022_H13N2 | North America | Gull |
| WCJ17385_A_Sanderling_DE_192_2022_H13N2 | North America | Shorebird |
| WCJ17511_A_Red_Knot_DE_1087_2022_H13N6 | North America | Shorebird |
| WEV00524_A_Franklins_gull_USA_D21-004347-2_MNAI1144_2017_H13N2 | North America | Gull |
| WEV00525_A_Franklins_gull_USA_D21-004347-3_MNAI1172_2017_H13N6 | North America | Gull |
| WEV00526_A_Franklins_gull_USA_D21-004347-4_MNAI1365_2017_H13N2 | North America | Gull |
