# Supplementary material for: Genetic Characterization and Phylogeographic Analysis of the First H13N6 Avian Influenza Virus Isolated from Vega Gull in South Korea
Source: Viruses. 2024 Feb 12;16(2):285. doi: 10.3390/v16020285 (PMC10891532; doi:10.3390/v16020285)
Supplement: Supplementary file 1 [file viruses-16-00285-s001.zip › Table S3 PB1 isolates.pdf]

Table S3. List of isolates used in this study for PB1 gene analysis

ISOLATE_ID	LOCATION	HOST
AEB66913_A_gull_Maryland_704_1977_H13N6	North America	Gull
ARX94017_A_black-headed_gull_Netherlands_1_2000_H13N8	Europe	Gull
ABB20100_A_shorebird_NJ_840_1986_H13N3	North America	Shorebird
ABB20117_A_laughing_gull_DE_554_1988_H13N3	North America	Gull
ABB20124_A_herring_gull_Delaware_660_1988_H13N6	North America	Gull
QBL97185_A_red_knot_Delaware_Bay_489_2010_H13N6	North America	Shorebird
ABB21769_A_gull_Minnesota_945_1980_H13N6	North America	Gull
ABB86519_A_herring_gull_DE_475_1986_H13N2	North America	Gull
ABB87342_A_shorebird_DE_68_2004_H13N9	North America	Shorebird
ABB87353_A_herring_gull_NJ_782_1986_H13N2	North America	Gull
ABB87819_A_laughing_gull_Delaware_Bay_2838_1987_H13N2	North America	Gull
ABI84720_A_turkey_Minnesota_1012_1990_H13N2	North America	Galliformes
ABI85207_A_shorebird_Delaware_224_1997_H13N6	North America	Shorebird
BAF37818_A_duck_Siberia_272_1998_H13N6	Asia	Anseriformes
BAF38380_A_duck_Siberia_272PF_1998_H13N6	Asia	Anseriformes
ACT84412_A_laughing_gull_New_Jersey_Sg-00485_2008_H13N9	North America	Gull
ACT84607_A_laughing_gull_New_Jersey_Sg-00559_2008_H13N9	North America	Gull
ACU15643_A_shorebird_Delaware_221_2006_H13N9	North America	Shorebird
ACV86838_A_black_headed_gull_Mongolia_1766_2006_H13N6	Asia	Gull
ACV86848_A_Mongolian_gull_Mongolia_401_2007_H13N6	Asia	Gull
ACZ45808_A_gull_Massachusetts_26_1980_H13N6	North America	Gull
ACZ45848_A_black-headed_gull_Astrakhan_65_1983_H13N6	Europe	Gull
ACZ45849_A_gull_Astrakhan_1846_1998_H13N6	Europe	Gull
ACZ45851_A_black-headed_gull_Astrakhan_458_1985_H13N6	Europe	Gull
ACZ45852_A_black-headed_gull_Astrakhan_44_1988_H13N6	Europe	Gull
ADF29863_A_glaucous_gull_Alaska_44199-006_2006_H13N9	North America	Gull
ADF29873_A_glaucous_gull_Alaska_44199-097_2006_H13N3	North America	Gull
AGL57556_A_ring-billed_gull_Georgia_AI00-2658_2000_H13N6	North America	Gull
AWV92021_A_ruddy_turnstone_New_Jersey_AI01-1407_2001_H13N6	North America	Shorebird
AXB35133_A_environment_New_Jersey_AI00-2413_2000_H13N6	North America	Environment
ADM07053_A_glaucous-winged_gull_Southcentral_Alaska_9JR0691R0_2009_H13N6	North America	Gull
ADM07064_A_glaucous-winged_gull_Southcentral_Alaska_9JR0738R0_2009_H13N6	North America	Gull
ADM07075_A_glaucous-winged_gull_Southcentral_Alaska_9JR0747R0_2009_H13N6	North America	Gull
ADM07097_A_glaucous-winged_gull_Southcentral_Alaska_9JR0781R0_2009_H13N6	North America	Gull
ADQ26648_A_black-headed_gull_Sweden_1_2005_H13N8	Europe	Gull
AEB71110_A_herring_gull_Mongolia_454_2008_H13N8	Asia	Gull
AFL56744_A_mallard_Korea_SH38-45_2010_H13N2	Asia	Anseriformes
AFP20878_A_ring-billed_gull_Quebec_02434-1_2009_H13N6	North America	Gull
AFP21000_A_black-legged_kittiwake_Quebec_02838-1_2009_H13N6	North America	Gull
AFX83980_A_ruddy_turnstone_Delaware_Bay_520_1988_H13N9	North America	Shorebird
AFX86017_A_shorebird_Delaware_Bay_424_2007_H13N9	North America	Shorebird
AFY06258_A_mallard-black_duck_hybrid_New_Brunswick_03736_2009_H13N6	North America	Anseriformes
AGL60143_A_ruddy_turnstone_New_Jersey_AI09-294_2009_H13N6	North America	Shorebird
AHA57186_A_seagull_Chile_5775_2009_H13N9	South America	Gull
AHJ58185_A_whale_Maine_328B_1984_H13N2	North America	Mammal
AHZ38836_A_black-headed_gull_Sweden_74342_2008_H13N8	Europe	Gull
AHZ39246_A_yellow-legged_gull_Georgia_1_2011_H13N6	Europe	Gull
AHZ39293_A_black-headed_gull_Georgia_1_2011_H13N8	Europe	Gull
AHZ39389_A_black-headed_gull_Republic_of_Georgia_2_2012_H13N6	Europe	Gull
AHZ39448_A_yellow-legged_gull_Republic_of_Georgia_2_2012_H13N6	Europe	Gull
AHZ39484_A_yellow-legged_gull_Georgia_1_2013_H13N8	Europe	Gull
AHZ39567_A_Armenian_gull_Republic_of_Georgia_1_2012_H13N6	Europe	Gull
AHZ39611_A_yellow-legged_gull_Republic_of_Georgia_6_2012_H13N6	Europe	Gull
AHZ39635_A_yellow-legged_gull_Republic_of_Georgia_5_2012_H13N6	Europe	Gull
AIN76260_A_herring_gull_Maine_ND0000047_2013_H13N6	North America	Gull
AKH14239_A_black-headed_gull_Netherlands_7_2009_H13N2	Europe	Gull
ALZ48199_A_glaucous-winged_gull_Southcentral_Alaska_11JR02474_2011_H13N6	North America	Gull
APC26578_A_black-headed_gull_Netherlands_9_2008_H13N8	Europe	Gull
AZR38843_A_laughing_gull_Delaware_Bay_451_2018_H13N6	North America	Gull
QDP69698_A_Glaucous-Winged_Gull_Southcentral_Alaska_17MB03638_2017_H13N2	North America	Gull
UEF69686_A_silver_gull_Tasmania_06-0349-105_2006_H13N6	Oceania	Gull
UZU89503_A_yellow-billed_teal_Chile_C38424_2018_H13N8	South America	Anseriformes
ALZ46756_A_ring-billed_gull_Massachusetts_12DC00060_2012_H13N6	North America	Gull
ALZ46780_A_gull_Massachusetts_12JR00671_2012_H13N6	North America	Gull
ALZ46987_A_ring-billed_gull_Massachusetts_13DC30736-1_2013_H13N8	North America	Gull
APC26542_A_black-headed_gull_Netherlands_23_2008_H13N8	Europe	Gull
APC26576_A_black-headed_gull_Netherlands_26_2014_H13N6	Europe	Gull
APC26580_A_black-headed_gull_Netherlands_21_2011_H13N8	Europe	Gull
APC26600_A_black-headed_gull_Netherlands_42_2012_H13N6	Europe	Gull
APC26608_A_black-headed_gull_Netherlands_23_2014_H13N2	Europe	Gull
APC26612_A_black-headed_gull_Netherlands_4_2007_H13N6	Europe	Gull
APC26618_A_black-headed_gull_Netherlands_12_2010_H13N2	Europe	Gull
APC26628_A_black-headed_gull_Netherlands_35_2011_H13N8	Europe	Gull
APC26636_A_black-headed_gull_Netherlands_100_2012_H13N6	Europe	Gull
APC26646_A_black-headed_gull_Netherlands_8_2010_H13N2	Europe	Gull
APC26648_A_black-headed_gull_Netherlands_17_2009_H13N3	Europe	Gull
APC26650_A_black-headed_gull_Netherlands_10_2007_H13N6	Europe	Gull
APC26680_A_black-headed_gull_Netherlands_6_2010_H13N8	Europe	Gull

APC26706_A_black-headed_gull_Netherlands_31_2009_H13N2	Europe	Gull
APC26712_A_black-headed_gull_Netherlands_9_2009_H13N2	Europe	Gull
APC26770_A_black-headed_gull_Netherlands_37_2009_H13N2	Europe	Gull
APC26784_A_black-headed_gull_Netherlands_92_2012_H13N6	Europe	Gull
APC26810_A_black-headed_gull_Netherlands_6_2014_H13N2	Europe	Gull
APC26824_A_black-headed_gull_Netherlands_1_2010_H13N6	Europe	Gull
APC26828_A_black-headed_gull_Netherlands_2_2012_H13N6	Europe	Gull
APC26846_A_black-headed_gull_Netherlands_10_2013_H13N8	Europe	Gull
APC26856_A_black-headed_gull_Netherlands_29_2014_H13N6	Europe	Gull
APC26906_A_black-headed_gull_Netherlands_7_2013_H13N8	Europe	Gull
APC26908_A_black-headed_gull_Netherlands_113_2012_H13N6	Europe	Gull
APC26938_A_black-headed_gull_Netherlands_102_2008_H13N8	Europe	Gull
APC26966_A_black-headed_gull_Netherlands_27_2009_H13N2	Europe	Gull
APC26988_A_black-headed_gull_Netherlands_39_2009_H13N6	Europe	Gull
APC27050_A_black-headed_gull_Netherlands_88_2012_H13N6	Europe	Gull
APC27058_A_black-headed_gull_Netherlands_11_2009_H13N6	Europe	Gull
APC27068_A_black-headed_gull_Netherlands_1_2009_H13N2	Europe	Gull
APC27072_A_black-headed_gull_Netherlands_20_2008_H13N8	Europe	Gull
APC27080_A_black-headed_gull_Netherlands_15_2014_H13N6	Europe	Gull
APC27082_A_black-headed_gull_Netherlands_87_2012_H13N6	Europe	Gull
APC27084_A_black-headed_gull_Netherlands_27_2014_H13N2	Europe	Gull
APC27101_A_black-headed_gull_Netherlands_105_2012_H13N6	Europe	Gull
APH09491_A_glaucous-winged_gull_Alaska_387_2013_H13N2	North America	Gull
APH09550_A_glaucous-winged_gull_Alaska_458_2013_H13N2	North America	Gull
APH09574_A_glaucous-winged_gull_Alaska_544_2013_H13N2	North America	Gull
APH09586_A_glaucous-winged_gull_Alaska_545_2013_H13N2	North America	Gull
APH09670_A_glaucous-winged_gull_Alaska_667_2013_H13N2	North America	Gull
APH09682_A_glaucous-winged_gull_Alaska_695_2013_H13N2	North America	Gull
APY16567_A_glaucous-winged_gull_Southcentral_Alaska_15MB01557_2015_H13N6	North America	Gull
APY16579_A_glaucous-winged_gull_Southcentral_Alaska_15MB01429_2015_H13N6	North America	Gull
APY16591_A_glaucous-winged_gull_Southcentral_Alaska_15MB01632_2015_H13N6	North America	Gull
APY16679_A_glaucous-winged_gull_Southcentral_Alaska_15MB02016_2015_H13N8	North America	Gull
ARX91971_A_yellow-legged_gull_Georgia_1_2011_H13N8	Europe	Gull
ARX91990_A_black-headed_gull_Netherlands_51_2008_H13N8	Europe	Gull
ARX92138_A_black-headed_gull_Netherlands_38_2009_H13N2	Europe	Gull
ARX92359_A_black-headed_gull_Netherlands_122_2012_H13N6	Europe	Gull
ARX92528_A_black-headed_gull_Netherlands_16_2014_H13N6	Europe	Gull
ARX93027_A_black-headed_gull_Netherlands_38_2008_H13N8	Europe	Gull
ARX93211_A_black-headed_gull_Netherlands_13_2014_H13N2	Europe	Gull
ARX93242_A_black-headed_gull_Netherlands_19_2012_H13N6	Europe	Gull
ARX93284_A_black-headed_gull_Netherlands_6_2013_H13N8	Europe	Gull
ARX93355_A_black-headed_gull_Netherlands_39_2011_H13N8	Europe	Gull
ARX93525_A_black-headed_gull_Netherlands_13_2012_H13N6	Europe	Gull
ARX93543_A_black-headed_gull_Netherlands_129_2012_H13N6	Europe	Gull
ARX93954_A_yellow-legged_gull_Georgia_4_2012_H13N8	Europe	Gull
ARX94830_A_black-headed_gull_Netherlands_6_2009_H13N2	Europe	Gull
AWV93207_A_ruddy_turnstone_New_Jersey_AI12-1737_2012_H13N6	North America	Shorebird
AXO65880_A_ring-billed_gull_Minnesota_CLMNAI1420_2017_H13N6	North America	Gull
AID48134_A_mallard_Dalian_DZ-137_2013_H13N6	Asia	Anseriformes
AKD00235_A_Eurasian_curlew_Liaoning_ZH-385_2014_H13N8	Asia	Shorebird
AKD00245_A_Eurasian_curlew_Liaoning_ZH-186_2014_H13N6	Asia	Shorebird
ARX94045_A_black-headed_gull_Netherlands_78_2012_H13N6	Europe	Gull
ARX94728_A_black-headed_gull_Netherlands_133_2012_H13N6	Europe	Gull
ARX94905_A_black-headed_gull_Netherlands_4_2011_H13N8	Europe	Gull
ASN77226_A_herring_gull_Massachusetts_A00080257_2006_H13N2	North America	Gull
ASO97741_A_black-tailed_gull_Weihai_115_2016_H13N2	Asia	Gull
BBB38797_A_duck_Hokkaido_W189_2006_H13N6	Asia	Anseriformes
AVO00177_A_laughing_gull_New_Jersey_UGAI17-2839_2017_H13N6	North America	Gull
AWF93143_A_environment_Chile_C7126_2016_H13N2	South America	Environment
AWF93399_A_brown-hooded_gull_Chile_C10256_2016_H13N2	South America	Gull
AWV92267_A_gull_New_Jersey_UGAI15-3416_2015_H13N6	North America	Gull
AWV92303_A_gull_New_Jersey_UGAI15-3767_2015_H13N3	North America	Gull
AWV94714_A_ruddy_turnstone_New_Jersey_UGAI14-1436_2014_H13N6	North America	Shorebird
AXB35145_A_herring_gull_New_Jersey_AI07-418_2007_H13N9	North America	Gull
AXF49022_A_Franklin_s_gull_Chile_C17421_2016_H13N9	South America	Gull
AXF49073_A_Franklin_s_gull_Chile_C20070_2016_H13N9	South America	Gull
AXF49078_A_Franklin_s_gull_Chile_C20086_2016_H13N9	South America	Gull
AXF49108_A_Franklin_s_gull_Chile_C20110_2016_H13N9	South America	Gull
AXF49114_A_Franklin_s_gull_Chile_C20118_2016_H13N9	South America	Gull
AXF49150_A_black_skimmer_Chile_C20057_2016_H13N8	South America	Shorebird
AXF49197_A_black_skimmer_Chile_C20108_2016_H13N9	South America	Shorebird
AXF49369_A_environment_Chile_C20128_2016_H13N9	South America	Environment
AXF49456_A_environment_Chile_C20168_2016_H13N9	South America	Environment
AXF49470_A_environment_Chile_C20173_2016_H13N9	South America	Environment
AXF49485_A_environment_Chile_C20175_2016_H13N9	South America	Environment
AXF49545_A_environment_Chile_C20180_2016_H13N9	South America	Environment
AXF49564_A_environment_Chile_C20182_2016_H13N9	South America	Environment
AXF49613_A_kelp_gull_Chile_C27733_2017_H13N8	South America	Gull
AXF49690_A_shorebird_Chile_C7037_2016_H13N2	South America	Shorebird
AXF49705_A_whimbrel_Chile_C20073_2016_H13N9	South America	Shorebird
AXF49774_A_white-backed_stilt_Chile_C20090_2016_H13N9	South America	Shorebird
AXK58069_A_ruddy_turnstone_Delaware_Bay_131_2004_H13N9	North America	Shorebird

AXK58279_A_kelp_gull_Delaware_Bay_304_2007_H13N9	North America	Gull
AXK58484_A_semipalmated_sandpiper_Delaware_Bay_142_2007_H13N9	North America	Shorebird
AXO65328_A_ring-billed_gull_Minnesota_OPMNAI0816_2017_H13N8	North America	Gull
AXO65340_A_ring-billed_gull_Minnesota_CLMNAI1320_2017_H13N8	North America	Gull
AXO65410_A_ring-billed_gull_Minnesota_CLMNAI0926_2017_H13N6	North America	Gull
AXO65458_A_ring-billed_gull_Minnesota_CLMNAI1025_2017_H13N6	North America	Gull
AXO65496_A_ring-billed_gull_Minnesota_CLMNAI0809_2017_H13N8	North America	Gull
AXO65529_A_ring-billed_gull_Minnesota_CLMNAI0891_2017_H13N6	North America	Gull
AXO65577_A_ring-billed_gull_Minnesota_CLMNAI0811_2017_H13N8	North America	Gull
AXO65800_A_ring-billed_gull_Minnesota_CLMNAI0955_2017_H13N8	North America	Gull
AXO65856_A_ring-billed_gull_Minnesota_CLMNAI1283_2017_H13N2	North America	Gull
AXO65868_A_ring-billed_gull_Minnesota_CLMNAI1301_2017_H13N2	North America	Gull
AXO65892_A_ring-billed_gull_Minnesota_CLMNAI1501_2017_H13N2	North America	Gull
AXO65904_A_ring-billed_gull_Minnesota_CLMNAI1529_2017_H13N6	North America	Gull
AXO65916_A_ring-billed_gull_Minnesota_CLMNAI1539_2017_H13N6	North America	Gull
AXY66758_A_Duck_China_Weihai_2017_H13N8	Asia	Anseriformes
QBM14646_A_herring_gull_Delaware_Bay_22_2014_H13N6	North America	Gull
QBM14725_A_laughing_gull_Delaware_Bay_39_2014_H13N6	North America	Gull
QBM14865_A_laughing_gull_Delaware_Bay_30_2014_H13N6	North America	Gull
QDP69674_A_Glaucous-Winged_Gull_Southcentral_Alaska_17MB03606_2017_H13N2	North America	Gull
QDP69686_A_Glaucous-Winged_Gull_Southcentral_Alaska_17MB03625_2017_H13N2	North America	Gull
QDP69784_A_Glaucous-Winged_Gull_Southcentral_Alaska_18MB00705_2018_H13N6	North America	Gull
QDP69832_A_Herring_Gull_Massachusetts_A00648685_2008_H13N2	North America	Gull
QDP69844_A_Herring_Gull_Massachusetts_A00648696_2008_H13N2	North America	Gull
QGR25244_A_Red_Knot_Delaware_Bay_3_2019_H13N6	North America	Shorebird
QOL20608_A_slender-billed_gull_Ukraine_CHBZ-11_2009_H13N2	Europe	Gull
QOL24242_A_shelduck_Ukraine_KT-9-2-11_2016_H13N6	Europe	Anseriformes
QTO29227_A_glaucous-winged_gull_Alaska_AK18-288_2018_H13N6	North America	Gull
QUD20222_A_Environmental_Santo_Domingo_GF89_2014_H13N2	South America	Environment
QUE37736_A_Glaucous-Winged_Gull_Alaska_20MB02831_2020_H13N6	North America	Gull
QUE37723_A_Glaucous-Winged_Gull_Alaska_20MB02182_2020_H13N6	North America	Gull
UEE12799_A_Kelp_gull_Peru_M8_2019_H13N6	South America	Gull
UNA44624_A_Red_Knot_Delaware_224_2021_H13N6	North America	Shorebird
UNA44784_A_Laughing_Gull_Delaware_294_2021_H13N6	North America	Gull
UZU88879_A_kelp_gull_Chile_C25097_2017_H13N9	South America	Gull
UZU88949_A_kelp_gull_Chile_C39002_2018_H13N2	South America	Gull
UZU88925_A_kelp_gull_Chile_C28937_2017_H13N8	South America	Gull
UZU89020_A_environment_Chile_C39659_2018_H13N2	South America	Environment
UZU89144_A_kelp_gull_Chile_C41225_2018_H13N6	South America	Gull
UZU89165_A_Franklin_s_gull_Chile_C42119_2018_H13N2	South America	Gull
UZU89268_A_environment_Chile_C39624_2018_H13N2	South America	Environment
UZU89316_A_southern_lapwing_Chile_C36480_2018_H13N8	South America	Other Charadriiformes
UZU89339_A_kelp_gull_Chile_C42093_2018_H13N2	South America	Gull
UZU89396_A_yellow-billed_teal_Chile_C37436_2018_H13N2	South America	Anseriformes
UZU89422_A_Franklin_s_gull_Chile_C33636_2017_H13N2	South America	Gull
WCJ14703_A_Great_black-backed_gull_DE_844_2022_H13N2	North America	Gull
WCJ14790_A_Laughing_Gull_DE_801_2022_H13N2	North America	Gull
WCJ14907_A_Laughing_Gull_DE_827_2022_H13N2	North America	Gull
WCJ17509_A_Red_Knot_DE_1087_2022_H13N6	North America	Shorebird
SKH13N6_PB1	SKH13N6	Gull
