# Supplementary material for: Genetic Characterization and Phylogeographic Analysis of the First H13N6 Avian Influenza Virus Isolated from Vega Gull in South Korea
Source: Viruses. 2024 Feb 12;16(2):285. doi: 10.3390/v16020285 (PMC10891532; doi:10.3390/v16020285)
Supplement: Supplementary file 1 [file viruses-16-00285-s001.zip › Table S4 PA isolates.pdf]

Table S4. List of isolates used in this study for PA gene analysis

ISOLATE_ID	LOCATION	HOST
AAA43118_A_gull_Maryland_704_1977_H13N6	North America	Gull
ABB20099_A_A_shorebird_NJ_840_1986_H13N3	North America	Shorebird
ABB20123_A_A_herring_gull_Delaware_660_1988_H13N6	North America	Gull
ABB21768_A_gull_Minnesota_945_1980_H13N6	North America	Gull
ABB86518_A_A_herring_gull_DE_475_1986_H13N2	North America	Gull
ABB87341_A_shorebird_DE_68_2004_H13N9	North America	Shorebird
ABB87352_A_herring_gull_NJ_782_1986_H13N2	North America	Gull
ABB87818_A_laughing_gull_Delaware_Bay_2838_1987_H13N2	North America	Gull
ABI84573_A_gull_Maryland_704_1977_H13N6	North America	Gull
ABI84719_A_turkey_Minnesota_1012_1990_H13N2	North America	Galliformes
ABI85206_A_shorebird_Delaware_224_1997_H13N6	North America	Shorebird
ACT84609_A_laughing_gull_New_Jersey_Sg-00559_2008_H13N9	North America	Gull
ACU15642_A_shorebird_Delaware_221_2006_H13N9	North America	Shorebird
ACV86837_A_black_headed_gull_Mongolia_1766_2006_H13N6	Asia	Gull
ACV86847_A_Mongolian_gull_Mongolia_401_2007_H13N6	Asia	Gull
ACZ46197_A_gull_Massachusetts_26_1980_H13N6	North America	Gull
ACZ46210_A_gull_Maryland_1824_1978_H13N9	North America	Gull
ACZ46238_A_black-headed_gull_Astrakhan_65_1983_H13N6	Europe	Gull
ACZ46239_A_blackhead_gull_Astrakhan_1421_1979_H13N6	Europe	Gull
ACZ46240_A_black-headed_gull_Astrakhan_458_1985_H13N6	Europe	Gull
ACZ46241_A_black-headed_gull_Astrakhan_44_1988_H13N6	Europe	Gull
ADD16612_A_great_black-backed_gull_Newfoundland_296_2008_H13N2	North America	Gull
ADF29832_A_A_glaucous_gull_Alaska_44199-104_2006_H13N9	North America	Gull
ADF29836_A_lesser_snow_goose_Alaska_44199-115_2006_H13N9	North America	Anseriformes
ADF29840_A_A_glaucous_gull_Alaska_44199-097_2006_H13N3	North America	Gull
ADF29842_A_A_glaucous_gull_Alaska_44199-006_2006_H13N9	North America	Gull
ADM07055_A_A_glaucous-winged_gull_Southcentral_Alaska_9JR0691R0_2009_H13N6	North America	Gull
ADM07066_A_A_glaucous-winged_gull_Southcentral_Alaska_9JR0738R0_2009_H13N6	North America	Gull
ADM07077_A_A_glaucous-winged_gull_Southcentral_Alaska_9JR0747R0_2009_H13N6	North America	Gull
ADM07088_A_A_glaucous-winged_gull_Southcentral_Alaska_9JR0769R0_2009_H13N6	North America	Gull
ADQ26647_A_black-headed_gull_Sweden_1_2005_H13N8	Europe	Gull
AEB66912_A_gull_Maryland_704_1977_H13N6	North America	Gull
AEB71109_A_herring_gull_Mongolia_454_2008_H13N8	Asia	Gull
AET74809_A_laughing_gull_Delaware_Bay_2838_1987_H13N2	North America	Gull
AFL56743_A_mallard_Korea_SH38-45_2010_H13N2	Asia	Anseriformes
AFP20877_A_ring-billed_gull_Quebec_02434-1_2009_H13N6	North America	Gull
AFX83979_A_ruddy_turnstone_Delaware_Bay_520_1988_H13N9	North America	Shorebird
AGB98190_A_glaucous-winged_gull_Southeastern_Alaska_9JR0822R0_2009_H13N6	North America	Gull
AGL57554_A_A_ring-billed_gull_Georgia_AI00-2658_2000_H13N6	North America	Gull
AGL58708_A_laughing_gull_New_Jersey_Sg-00485_2008_H13N9	North America	Gull
AGL60141_A_ruddy_turnstone_New_Jersey_AI09-294_2009_H13N6	North America	Shorebird
AHA57188_A_seagull_Chile_5775_2009_H13N9	South America	Gull
AHJ58187_A_whale_Maine_328B_1984_H13N2	North America	Mammal
AHZ38834_A_black-headed_gull_Sweden_74342_2008_H13N8	Europe	Gull
AHZ39232_A_black-headed_gull_Republic_of_Georgia_7_2011_H13N6	Europe	Gull
AHZ39291_A_black-headed_gull_Georgia_1_2011_H13N8	Europe	Gull
AHZ39351_A_black-headed_gull_Georgia_6_2011_H13N8	Europe	Gull
AHZ39446_A_yellow-legged_gull_Republic_of_Georgia_2_2012_H13N6	Europe	Gull
AHZ39458_A_yellow-legged_gull_Republic_of_Georgia_3_2012_H13N6	Europe	Gull
AHZ39482_A_yellow-legged_gull_Georgia_1_2013_H13N8	Europe	Gull
AHZ39505_A_yellow-legged_gull_Georgia_2_2013_H13N8	Europe	Gull
AHZ39553_A_black-headed_gull_Georgia_9_2011_H13N8	Europe	Gull
AHZ39565_A_Armenian_gull_Republic_of_Georgia_1_2012_H13N6	Europe	Gull
AHZ39577_A_common_snipe_Republic_of_Georgia_1_2012_H13N2	Europe	Shorebird
AHZ39633_A_yellow-legged_gull_Republic_of_Georgia_5_2012_H13N6	Europe	Gull
AID48135_A_mallard_Dalian_DZ-137_2013_H13N6	Asia	Anseriformes
AIN76262_A_herring_gull_Maine_ND0000047_2013_H13N6	North America	Gull
AKD00236_A_Eurasian_curlew_Liaoning_ZH-385_2014_H13N8	Asia	Shorebird
AKD00246_A_Eurasian_curlew_Liaoning_ZH-186_2014_H13N6	Asia	Shorebird
AKH14241_A_black-headed_gull_Netherlands_7_2009_H13N2	Europe	Gull
ALZ46778_A_gull_Massachusetts_12JR00671_2012_H13N6	North America	Gull
ALZ46985_A_ring-billed_gull_Massachusetts_13DC30736-1_2013_H13N8	North America	Gull
ALZ48197_A_glaucous-winged_gull_Southcentral_Alaska_11JR02474_2011_H13N6	North America	Gull
APC30503_A_black-headed_gull_Netherlands_1_2010_H13N6	Europe	Gull
APC30642_A_black-headed_gull_Netherlands_2_2007_H13N6	Europe	Gull
APC30658_A_black-headed_gull_Netherlands_27_2014_H13N2	Europe	Gull
APC30691_A_black-headed_gull_Netherlands_1_2014_H13N2	Europe	Gull
APC30743_A_black-headed_gull_Netherlands_6_2010_H13N8	Europe	Gull
APC30855_A_black-headed_gull_Netherlands_55_2008_H13N8	Europe	Gull
APC30910_A_black-headed_gull_Netherlands_23_2014_H13N2	Europe	Gull
APC30932_A_black-headed_gull_Netherlands_20_2008_H13N8	Europe	Gull
APC31012_A_black-headed_gull_Netherlands_88_2012_H13N6	Europe	Gull
APC31019_A_black-headed_gull_Netherlands_82_2012_H13N6	Europe	Gull
APC31117_A_black-headed_gull_Netherlands_10_2013_H13N8	Europe	Gull
APC31127_A_black-headed_gull_Netherlands_8_2010_H13N2	Europe	Gull
APC31230_A_black-headed_gull_Netherlands_2_2011_H13N8	Europe	Gull
APC31234_A_black-headed_gull_Netherlands_11_2014_H13N6	Europe	Gull
APC31411_A_black-headed_gull_Netherlands_52_2012_H13N6	Europe	Gull

APC31443_A_black-headed_gull_Netherlands_134_2012_H13N6	Europe	Gull
APC31451_A_black-headed_gull_Netherlands_17_2009_H13N3	Europe	Gull
APC31677_A_black-headed_gull_Netherlands_12_2010_H13N2	Europe	Gull
APC32034_A_black-headed_gull_Netherlands_3_2014_H13N6	Europe	Gull
APC32074_A_black-headed_gull_Netherlands_106_2012_H13N6	Europe	Gull
APC32210_A_black-headed_gull_Netherlands_10_2014_H13N6	Europe	Gull
APC32215_A_black-headed_gull_Netherlands_2_2012_H13N6	Europe	Gull
APC32322_A_black-headed_gull_Netherlands_83_2012_H13N6	Europe	Gull
APC32325_A_black-headed_gull_Netherlands_113_2012_H13N6	Europe	Gull
APC32352_A_black-headed_gull_Netherlands_18_2011_H13N8	Europe	Gull
APC32450_A_black-headed_gull_Netherlands_31_2009_H13N2	Europe	Gull
APC32477_A_black-headed_gull_Netherlands_39_2009_H13N6	Europe	Gull
APC32602_A_black-headed_gull_Netherlands_10_2010_H13N8	Europe	Gull
APC32784_A_black-headed_gull_Netherlands_105_2012_H13N6	Europe	Gull
APC32858_A_black-headed_gull_Netherlands_5_2014_H13N6	Europe	Gull
APC32866_A_black-headed_gull_Netherlands_27_2009_H13N2	Europe	Gull
APC32944_A_black-headed_gull_Netherlands_9_2008_H13N8	Europe	Gull
APC33184_A_black-headed_gull_Netherlands_9_2009_H13N2	Europe	Gull
APH09493_A_glaucous-winged_gull_Alaska_387_2013_H13N2	North America	Gull
APH09528_A_glaucous-winged_gull_Alaska_414_2013_H13N2	North America	Gull
APH09552_A_glaucous-winged_gull_Alaska_458_2013_H13N2	North America	Gull
APH09588_A_glaucous-winged_gull_Alaska_545_2013_H13N2	North America	Gull
APH09672_A_glaucous-winged_gull_Alaska_667_2013_H13N2	North America	Gull
APY16425_A_glaucous-winged_gull_Southcentral_Alaska_15MB01610_2015_H13N6	North America	Gull
APY16565_A_glaucous-winged_gull_Southcentral_Alaska_15MB01557_2015_H13N6	North America	Gull
APY16577_A_glaucous-winged_gull_Southcentral_Alaska_15MB01429_2015_H13N6	North America	Gull
ARX91508_A_black-headed_gull_Netherlands_7_2008_H13N8	Europe	Gull
ARX91631_A_black-headed_gull_Netherlands_3_2009_H13N2	Europe	Gull
ARX91800_A_A_yellow-legged_gull_Georgia_1_2011_H13N8	Europe	Gull
ARX91805_A_black-headed_gull_Netherlands_67_2008_H13N8	Europe	Gull
ARX92100_A_black-headed_gull_Netherlands_26_2011_H13N8	Europe	Gull
ARX92118_A_black-headed_gull_Netherlands_49_2012_H13N6	Europe	Gull
ARX92236_A_yellow-legged_gull_Georgia_4_2012_H13N8	Europe	Gull
ARX92421_A_black-headed_gull_Netherlands_131_2012_H13N6	Europe	Gull
ARX92428_A_black-headed_gull_Netherlands_9_2013_H13N8	Europe	Gull
ARX92578_A_black-headed_gull_Netherlands_3_2008_H13N8	Europe	Gull
ARX92684_A_black-headed_gull_Netherlands_1_2012_H13N6	Europe	Gull
ARX93336_A_black-headed_gull_Netherlands_93_2008_H13N8	Europe	Gull
ARX93445_A_black-headed_gull_Netherlands_66_2008_H13N8	Europe	Gull
ARX93830_A_black-headed_gull_Netherlands_17_2012_H13N6	Europe	Gull
ARX93897_A_black-headed_gull_Netherlands_44_2008_H13N8	Europe	Gull
ARX93919_A_black-headed_gull_Netherlands_12_2011_H13N8	Europe	Gull
ARX94048_A_black-headed_gull_Netherlands_97_2012_H13N6	Europe	Gull
ARX94076_A_black-headed_gull_Netherlands_14_2011_H13N8	Europe	Gull
ARX94086_A_black-headed_gull_Netherlands_2_2008_H13N8	Europe	Gull
ARX94163_A_black-headed_gull_Netherlands_16_2014_H13N6	Europe	Gull
ARX94193_A_black-headed_gull_Netherlands_52_2008_H13N8	Europe	Gull
ARX94256_A_black-headed_gull_Netherlands_24_2008_H13N8	Europe	Gull
ARX94380_A_black-headed_gull_Netherlands_3_2011_H13N8	Europe	Gull
ARX94385_A_black-headed_gull_Netherlands_95_2012_H13N6	Europe	Gull
ARX94408_A_black-headed_gull_Netherlands_2_2010_H13N8	Europe	Gull
ARX94514_A_black-headed_gull_Netherlands_38_2011_H13N8	Europe	Gull
ARX94723_A_black-headed_gull_Netherlands_6_2013_H13N8	Europe	Gull
ARX94956_A_black-headed_gull_Netherlands_1_2000_H13N8	Europe	Gull
ASN77224_A_herring_gull_Massachusetts_A00080257_2006_H13N2	North America	Gull
ASO97742_A_black-tailed_gull_Weihai_115_2016_H13N2	Asia	Gull
ASO97752_A_black-tailed_gull_Weihai_17_2016_H13N8	Asia	Gull
AVO00175_A_laughing_gull_New_Jersey_UGAI17-2839_2017_H13N6	North America	Gull
AWF93201_A_A_environment_Chile_C7144_2016_H13N2	South America	Environment
AWF93226_A_A_environment_Chile_C7125_2016_H13N2	South America	Environment
AWF93300_A_A_whimbrel_Chile_C10834_2016_H13N2	South America	Shorebird
AWF93404_A_A_environment_Chile_C7039_2016_H13N2	South America	Environment
AWF93480_A_brown-hooded_gull_Chile_C10765_2016_H13N2	South America	Gull
AWF93497_A_A_whimbrel_Chile_C12523_2016_H13N2	South America	Shorebird
AWV92019_A_ruddy_turnstone_New_Jersey_AI01-1407_2001_H13N6	North America	Shorebird
AWV92301_A_gull_New_Jersey_UGAI15-3767_2015_H13N3	North America	Gull
AWV93205_A_ruddy_turnstone_New_Jersey_AI12-1737_2012_H13N6	North America	Shorebird
AXB35131_A_A_environment_New_Jersey_AI00-2413_2000_H13N6	North America	Environment
AXB35143_A_herring_gull_New_Jersey_AI07-418_2007_H13N9	North America	Gull
AXE75371_A_A_environment_Chile_C20076_2016_H13N9	South America	Environment
AXF49024_A_A_Franklins_gull_Chile_C17421_2016_H13N9	South America	Gull
AXF49041_A_A_Franklins_gull_Chile_C20061_2016_H13N9	South America	Gull
AXF49060_A_Franklins_gull_Chile_C20069_2016_H13N9	South America	Gull
AXF49064_A_Franklins_gull_Chile_C20070_2016_H13N9	South America	Gull
AXF49117_A_Franklins_gull_Chile_C20118_2016_H13N9	South America	Gull
AXF49127_A_Franklins_gull_Chile_C20149_2016_H13N9	South America	Gull
AXF49190_A_black_skimmer_Chile_C20100_2016_H13N9	South America	Shorebird
AXF49310_A_elegant_tern_Chile_C20093_2016_H13N9	South America	Other Charadriiformes
AXF49346_A_environment_Chile_C20125_2016_H13N9	South America	Environment
AXF49393_A_environment_Chile_C20143_2016_H13N9	South America	Environment
AXF49516_A_environment_Chile_C20177_2016_H13N9	South America	Environment
AXF49610_A_kelp_gull_Chile_C27733_2017_H13N8	South America	Gull

AXF49625_A_kelp_gull_Chile_C8594_2016_H13N2	South America	Gull
AXF49629_A_kelp_gull_Chile_C8595_2016_H13N2	South America	Gull
AXF49674_A_kelp_gull_Chile_C8609_2016_H13N2	South America	Gull
AXF49716_A_whimbrel_Chile_C20075_2016_H13N9	South America	Shorebird
AXK58839_A_ruddy_turnstone_Delaware_Bay_131_2004_H13N9	North America	Shorebird
AXO65326_A_ring-billed_gull_Minnesota_OPMNAI0816_2017_H13N8	North America	Gull
AXO65338_A_ring-billed_gull_Minnesota_CLMNAI1320_2017_H13N8	North America	Gull
AXO65350_A_ring-billed_gull_Minnesota_OPMNAI0926_2017_H13N6	North America	Gull
AXO65456_A_ring-billed_gull_Minnesota_CLMNAI1025_2017_H13N6	North America	Gull
AXO65527_A_ring-billed_gull_Minnesota_CLMNAI0891_2017_H13N6	North America	Gull
AXO65637_A_ring-billed_gull_Minnesota_OPMNAI0800_2017_H13N8	North America	Gull
AXO65666_A_ring-billed_gull_Minnesota_CLMNAI1199_2017_H13N2	North America	Gull
AXO65774_A_ring-billed_gull_Minnesota_CLMNAI0939_2017_H13N6	North America	Gull
AXO65878_A_ring-billed_gull_Minnesota_CLMNAI1420_2017_H13N6	North America	Gull
AXO65890_A_ring-billed_gull_Minnesota_CLMNAI1501_2017_H13N2	North America	Gull
AXO65902_A_ring-billed_gull_Minnesota_CLMNAI1529_2017_H13N6	North America	Gull
AXO65914_A_ring-billed_gull_Minnesota_CLMNAI1539_2017_H13N6	North America	Gull
AXY66760_A_Duck_China_Weihai_2017_H13N8	Asia	Anseriformes
AZR38852_A_laughing_gull_Delaware_Bay_451_2018_H13N6	North America	Gull
AZV03313_A_kelp_gull_Delaware_Bay_441_2018_H13N6	North America	Gull
BAF37820_A_A_Duck_Siberia_272_1998_H13N6	Asia	Anseriformes
BBB38799_A_A_Duck_Hokkaido_W189_2006_H13N6	Asia	Anseriformes
CCH50637_A_common_gull_Norway_10_1313_2009_H13N2	Europe	Gull
QBL97183_A_Red_Knot_Delaware_Bay_489_2010_H13N6	North America	Shorebird
QBM14651_A_herring_gull_Delaware_Bay_22_2014_H13N6	North America	Gull
QBM14723_A_laughing_gull_Delaware_Bay_39_2014_H13N6	North America	Gull
QDP69636_A_glaucous_Gull_Northern_Alaska_18AR00289_2018_H13N6	North America	Gull
QDP69672_A_glaucous-Winged_Gull_Southcentral_Alaska_17MB03606_2017_H13N2	North America	Gull
QDP69684_A_glaucous-Winged_Gull_Southcentral_Alaska_17MB03625_2017_H13N2	North America	Gull
QDP69696_A_glaucous-Winged_Gull_Southcentral_Alaska_17MB03638_2017_H13N2	North America	Gull
QDP69720_A_glaucous-Winged_Gull_Southcentral_Alaska_18MB00667_2018_H13N6	North America	Gull
QDP69732_A_glaucous-Winged_Gull_Southcentral_Alaska_18MB00676_2018_H13N6	North America	Gull
QDP69746_A_glaucous-Winged_Gull_Southcentral_Alaska_18MB00688_2018_H13N6	North America	Gull
QDP69806_A_glaucous-Winged_Gull_Southcentral_Alaska_18MB03351_2018_H13N6	North America	Gull
QDP69830_A_herring_gull_Massachusetts_A00648685_2008_H13N2	North America	Gull
QDP69842_A_herring_gull_Massachusetts_A00648696_2008_H13N2	North America	Gull
QGR25240_A_Red_Knot_Delaware_Bay_3_2019_H13N6	North America	Shorebird
QOL20606_A_slender-billed_gull_Ukraine_CHBZ-11_2009_H13N2	Europe	Gull
QOL24244_A_shelduck_Ukraine_KT-9-2-11_2016_H13N6	Europe	Anseriformes
QTO29229_A_glaucous-winged_gull_Alaska_AK18-288_2018_H13N6	North America	Gull
QTO33858_A_Red_Knot_Delaware_Bay_605_2020_H13N6	North America	Shorebird
QTO34595_A_Red_Knot_Delaware_Bay_420_2020_H13N6	North America	Shorebird
QUD20220_A_environmental_Santo_Domingo_GF89_2014_H13N2	South America	Environment
QUE37719_A_glaucous-Winged_Gull_Alaska_20MB02182_2020_H13N6	North America	Gull
QUE37738_A_glaucous-Winged_Gull_Alaska_20MB02831_2020_H13N6	North America	Gull
QUE37802_A_glaucous-Winged_Gull_Alaska_20MB02498_2020_H13N6	North America	Gull
QUE37823_A_glaucous-Winged_Gull_Alaska_20MB02836_2020_H13N6	North America	Gull
QUE37852_A_glaucous-Winged_Gull_Alaska_20MB02534_2020_H13N6	North America	Gull
QUE37873_A_glaucous-Winged_Gull_Alaska_20MB02820_2020_H13N6	North America	Gull
SKH13N6_PA	SKH13N6	Gull
UEE12801_A_kelp_gull_Peru_M8_2019_H13N6	South America	Gull
UEF69688_A_silver_gull_Tasmania_06-0349-105_2006_H13N6	Oceania	Gull
UNA44622_A_Red_Knot_Delaware_224_2021_H13N6	North America	Shorebird
UNA44782_A_laughing_gull_Delaware_294_2021_H13N6	North America	Gull
UZU88881_A_kelp_gull_Chile_C25097_2017_H13N9	South America	Gull
UZU88951_A_kelp_gull_Chile_C39002_2018_H13N2	South America	Gull
UZU89022_A_environment_Chile_C39659_2018_H13N2	South America	Environment
UZU89087_A_kelpgull_Chile_C24014_2017_H13N9	South America	Gull
UZU89146_A_kelp_gull_Chile_C41225_2018_H13N6	South America	Gull
UZU89223_A_kelp_gull_Chile_C41186_2018_H13N8	South America	Gull
UZU89270_A_environment_Chile_C39624_2018_H13N2	South America	Environment
UZU89318_A_southern_lapwing_Chile_C36480_2018_H13N8	South America	Other Charadriiformes
UZU89424_A_Franklins_gull_Chile_C33636_2017_H13N2	South America	Gull
UZU89505_A_yellow-billed_teal_Chile_C38424_2018_H13N8	South America	Anseriformes
WCJ14708_A_great_black-backed_gull_DE_844_2022_H13N2	North America	Gull
WCJ14788_A_laughing_gull_DE_801_2022_H13N2	North America	Gull
WCJ14905_A_laughing_gull_DE_827_2022_H13N2	North America	Gull
WCJ17359_A_laughing_gull_DE_809_2022_H13N2	North America	Gull
WCJ17507_A_Red_Knot_DE_1087_2022_H13N6	North America	Shorebird
WEV00533_A_Franklins_gull_USA_D21-004347-2_MNAI1144_2017_H13N2	North America	Gull
WEV00535_A_Franklins_gull_USA_D21-004347-3_MNAI1172_2017_H13N6	North America	Gull
WEV00537_A_Franklins_gull_USA_D21-004347-4_MNAI1365_2017_H13N2	North America	Gull
