# Supplementary material for: Genetic Characterization and Phylogeographic Analysis of the First H13N6 Avian Influenza Virus Isolated from Vega Gull in South Korea
Source: Viruses. 2024 Feb 12;16(2):285. doi: 10.3390/v16020285 (PMC10891532; doi:10.3390/v16020285)
Supplement: Supplementary file 1 [file viruses-16-00285-s001.zip › Table S5 HA isolates.pdf]

Table S5. List of isolates used in this study for HA gene analysis

ISOLATE_ID	LOCATION	HOST
AAA43213_A_black-headed_gull_Astrakhan_227_1984_H13N6	Europe	Gull
AAA43214_A_ring-billed_gull_Maryland_704_1977_H13N6	North America	Gull
AAA43215_A_pilot_whale_Maine_328_HN_1984_H13N2	North America	Mammal
AAV91212_A_black-headed_gull_Netherlands_1_2000_H13N8	Europe	Gull
AAV91213_A_black-headed_gull_Sweden_1_1999_H13N6	Europe	Gull
ABB86511_A_herring_gull_DE_475_1986_H13N2	North America	Gull
ABB87334_A_shorebird_DE_68_2004_H13N9	North America	Shorebird
ABB87345_A_herring_gull_NJ_782_1986_H13N2	North America	Gull
ABB87811_A_laughing_gull_Delaware_Bay_2838_1987_H13N2	North America	Gull
ABI84452_A_herring_gull_Delaware_660_1988_H13N6	North America	Gull
ABI84566_A_gull_Maryland_704_1977_H13N6	North America	Gull
ABI84601_A_gull_Minnesota_945_1980_H13N6	North America	Gull
ACA48469_A_great_black-headed_gull_Astrakhan_1420_1979_H13N2	Europe	Gull
ACA48471_A_great_black-headed_gull_Astrakhan_591_1982_H13N2	Europe	Gull
ACD40325_A_kelp_gull_Argentina_LDC4_2006_H13N9	South America	Gull
ACE78933_A_Larus_ichthyaetus_Astrakhan_10_1988_H13N6	Europe	Gull
ACE78934_A_Larus_ichthyaetus_Astrakhan_75_1983_H13N2	Europe	Gull
ACE78935_A_Larus_minutus_Astrakhan_3357_2002_H13N6	Europe	Gull
ACE78942_A_black-headed_gull_Astrakhan_44_1988_H13N6	Europe	Gull
ACE78983_A_gull_Astrakhan_1846_1998_H13N6	Europe	Gull
ACE78984_A_black-headed_gull_Astrakhan_65_1983_H13N6	Europe	Gull
ACF04701_A_gull_Astrakhan_226_1984_H13N6	Europe	Gull
ACF04702_A_gull_Astrakhan_998_1990_H13N6	Europe	Gull
ACF04703_A_gull_Astrakhan_3483_2002_H13N6	Europe	Gull
ACF04704_A_gull_Astrakhan_1314_1979__H13N2	Europe	Gull
ACF04705_A_gull_Astrakhan_176_1986_H13N2	Europe	Gull
ACF04706_A_gull_Astrakhan_1818_1998_H13N6	Europe	Gull
ACU15632_A_shorebird_Delaware_221_2006_H13N9	North America	Shorebird
ACV86830_A_black_headed_gull_Mongolia_1766_2006_H13N6	Asia	Gull
ACV86840_A_Mongolian_gull_Mongolia_401_2007__H13N6	Asia	Gull
ACV86850_A_Mongolian_gull_Mongolia_405_2007_H13N6	Asia	Gull
ADB46159_A_American_white_pelican_Minnesota_AI-07-1819_2007_H13N9	North America	Pelecaniformes
ADB46161_A_American_white_pelican_Minnesota_Sg-0611_2008_H13N9	North America	Pelecaniformes
ADD92017_A_great_black-headed_gull_Atyrau_743_2004_H13N6	Asia	Gull
ADD92020_A_great_black-headed_gull_Atyrau_773_2004_H13N6	Asia	Gull
ADF29822_A_glaucous_gull_Alaska_44199-097_2006_H13N3	North America	Gull
ADM07067_A_glaucous-winged_gull_Southcentral_Alaska_9JR0738R0_2009_H13N6	North America	Gull
ADM07078_A_glaucous-winged_gull_Southcentral_Alaska_9JR0747R0_2009_H13N6	North America	Gull
ADM07089_A_glaucous-winged_gull_Southcentral_Alaska_9JR0769R0_2009_H13N6	North America	Gull
ADQ26640_A_black-headed_gull_Sweden_1_2005_H13N8	Europe	Gull
AEB71102_A_herring_gull_Mongolia_454_2008_H13N8	Asia	Gull
AFL56747_A_mallard_Korea_SH38-45_2010_H13N2	Asia	Anseriformes
AFP20992_A_black-legged_kittiwake_Quebec_02838-1_2009_H13N6	North America	Gull
AFP21003_A_hooded_merganser_New_Brunswick_03750_2009_H13N6	North America	Anseriformes
AFX83972_A_ruddy_turnstone_Delaware_Bay_520_1988_H13N9	North America	Shorebird
AFX86009_A_shorebird_Delaware_Bay_424_2007_H13N9	North America	Shorebird
AGB51312_A_gull_Maryland_704_1977_H13N6	North America	Gull
AGB98183_A_glaucous-winged_gull_Southeastern_Alaska_9JR0822R0_2009_H13N6	North America	Gull
AGK42180_A_laughing_gull_New_Jersey_Sg-00568_2008_H13N9	North America	Gull
AGL57547_A_ring-billed_gull_Georgia_AI00-2658_2000_H13N6	North America	Gull
AGL58701_A_laughing_gull_New_Jersey_Sg-00485_2008_H13N9	North America	Gull
AGL60134_A_ruddy_turnstone_New_Jersey_AI09-294_2009_H13N6	North America	Shorebird
AHJ58189_A_whale_Maine_328B_1984_H13N2	North America	Mammal
AHZ38827_A_black-headed_gull_Sweden_74342_2008_H13N8	Europe	Gull
AHZ39225_A_black-headed_gull_Republic_of_Georgia_7_2011_H13N6	Europe	Gull
AHZ39237_A_yellow-legged_gull_Georgia_1_2011_H13N6	Europe	Gull
AHZ39284_A_black-headed_gull_Georgia_1_2011_H13N8	Europe	Gull
AHZ39308_A_black-headed_gull_Georgia_3_2011_H13N8	Europe	Gull
AHZ39380_A_black-headed_gull_Republic_of_Georgia_2_2012_H13N6	Europe	Gull
AHZ39439_A_yellow-legged_gull_Republic_of_Georgia_2_2012_H13N6	Europe	Gull
AHZ39451_A_yellow-legged_gull_Republic_of_Georgia_3_2012_H13N6	Europe	Gull
AHZ39475_A_yellow-legged_gull_Georgia_1_2013_H13N8	Europe	Gull
AHZ39546_A_black-headed_gull_Georgia_9_2011_H13N8	Europe	Gull
AHZ39602_A_yellow-legged_gull_Republic_of_Georgia_6_2012_H13N6	Europe	Gull
AIX94903_A_gull_Maryland_16_1985_H13N2	North America	Gull
ALZ46747_A_ring-billed_gull_Massachusetts_12DC00060_2012_H13N6	North America	Gull
ALZ46771_A_gull_Massachusetts_12JR00671_2012_H13N6	North America	Gull
ALZ46978_A_ring-billed_gull_Massachusetts_13DC30736-1_2013_H13N8	North America	Gull
AMP44543_A_slender-billed_gull_CHBZ_11_2009_H13N2	Europe	Gull
ANT47310_A_herring_gull_Finland_9875_2005_H13	Europe	Gull
ANT47311_A_herring_gull_Finland_9330_2007_H13	Europe	Gull
ANT47312_A_herring_gull_Finland_9611_2007_H13	Europe	Gull
APY16558_A_glaucous-winged_gull_Southcentral_Alaska_15MB01557_2015_H13N6	North America	Gull
APY16570_A_glaucous-winged_gull_Southcentral_Alaska_15MB01429_2015_H13N6	North America	Gull
APY16582_A_glaucous-winged_gull_Southcentral_Alaska_15MB01632_2015_H13N6	North America	Gull

APY16670_A_glaucous-winged_gull_Southcentral_Alaska_15MB02016_2015_H13N8	North America	Gull
BAA14338_A_gull_Maryland_704_1977_H13N6	North America	Gull
BAF37821_A_duck_Siberia_272_1998_H13N6	Asia	Anseriformes
BAF38383_A_duck_Siberia_272PF_1998_H13N6	Asia	Anseriformes
BAF46906_A_gull_Maryland_704_1977_H13N6	North America	Gull
BAN14686_A_duck_Hokkaido_WZ68_2012_H13N2	Asia	Anseriformes
CAQ77191_A_herring_gull_Norway_10_2336_2006_H13N6	Europe	Gull
CCH50638_A_common_gull_Norway_10_1313_2009_H13N2	Europe	Gull
SKH13N6_HA	SKH13N6	Gull
