# Supplementary material for: Genetic Characterization and Phylogeographic Analysis of the First H13N6 Avian Influenza Virus Isolated from Vega Gull in South Korea
Source: Viruses. 2024 Feb 12;16(2):285. doi: 10.3390/v16020285 (PMC10891532; doi:10.3390/v16020285)
Supplement: Supplementary file 1 [file viruses-16-00285-s001.zip › Table S6 NP isolates.pdf]

Table S6. List of isolates used in this study for NP gene analysis

ISOLATE_ID	LOCATION	HOST
AAA43460_A_gull_Massachusetts_26_1980_H13N6	North America	Gull
AAA43461_A_gull_Astrakhan_227_1984_H13N6	Europe	Gull
AAA43463_A_gull_Maryland_1824_1978_H13N9	North America	Gull
AAA43464_A_gull_Maryland_1815_1979_H13N6	North America	Gull
AAA43472_A_gull_Minnesota_945_1980_H13N6	North America	Gull
ABB20096_A_shorebird_NJ_840_1986_H13N3	North America	Shorebird
ABB20113_A_laughing_gull_DE_554_1988_H13N3	North America	Gull
ABB21765_A_gull_Minnesota_945_1980_H13N6	North America	Gull
ABB86515_A_herring_gull_DE_475_1986_H13N2	North America	Gull
ABB87338_A_shorebird_DE_68_2004_H13N9	North America	Shorebird
ABB87349_A_herring_gull_NJ_782_1986_H13N2	North America	Gull
ABB87815_A_laughing_gull_Delaware_Bay_2838_1987_H13N2	North America	Gull
ABI84716_A_turkey_Minnesota_1012_1990_1990_H13N2	North America	Galliformes
ACU15638_A_shorebird_Delaware_221_2006_H13N9	North America	Shorebird
ACV86834_A_black_headed_gull_Mongolia_1766_2006_H13N6	Asia	Gull
ACV86844_A_Mongolian_gull_Mongolia_401_2007_H13N6	Asia	Gull
ACZ46614_A_black-headed_gull_Astrakhan_65_1983_H13N6	Europe	Gull
ACZ46616_A_blackhead_gull_Astrakhan_1421_1979_H13N6	Europe	Gull
ACZ46617_A_black-headed_gull_Astrakhan_458_1985_H13N6	Europe	Gull
ACZ46618_A_black-headed_gull_Astrakhan_44_1988_H13N6	Europe	Gull
ADD92025_A_great_black-headed_gull_Atyrau_743_2004_H13N6	Asia	Gull
ADD92026_A_great_black-headed_gull_Atyrau_744_2004_H13N6	Asia	Gull
ADD92028_A_great_black-headed_gull_Atyrau_773_2004_H13N6	Asia	Gull
ADQ26644_A_black-headed_gull_Sweden_1_2005_2005_H13N8	Europe	Gull
AEB66909_A_gull_Maryland_704_1977_H13N6	North America	Gull
AEB71108_A_herring_gull_Mongolia_454_2008_H13N8	Asia	Gull
AEK65264_A_silver_gull_Tasmania_062_2006_H13N6	Oceania	Gull
AFL56751_A_mallard_Korea_SH38-45_2010_H13N2	Asia	Anseriformes
AFP20874_A_ring-billed_gull_Quebec_02434-1_2009_H13N6	North America	Gull
AFW90047_A_gull_Maryland_1824_1978_H13N9	North America	Gull
AFX83976_A_ruddy_turnstone_Delaware_Bay_520_1988_H13N9	North America	Shorebird
AGB98187_A_glaucous-winged_gull_Southeastern_Alaska_9JR0822R0_2009_H13N6	North America	Gull
AGL57551_A_ring-billed_gull_Georgia_AI00-2658_2000_H13N6	North America	Gull
AHA57191_A_seagull_Chile_5775_2009_H13N9	South America	Gull
AHJ58190_A_whale_Maine_328B_1984_H13N2	North America	Mammal
AHZ39229_A_black-headed_gull_Republic_of_Georgia_7_2011_H13N6	Europe	Gull
AHZ39241_A_yellow-legged_gull_Georgia_1_2011_H13N6	Europe	Gull
AHZ39372_A_black-headed_gull_Republic_of_Georgia_1_2012_H13N6	Europe	Gull
AHZ39384_A_black-headed_gull_Republic_of_Georgia_2_2012_H13N6	Europe	Gull
AHZ39479_A_yellow-legged_gull_Georgia_1_2013_H13N8	Europe	Gull
AHZ39550_A_black-headed_gull_Georgia_9_2011_H13N8	Europe	Gull
AHZ39562_A_Armenian_gull_Republic_of_Georgia_1_2012_H13N6	Europe	Gull
AID48137_A_mallard_Dalian_DZ-137_2013_H13N6	Asia	Anseriformes
AKD00238_A_Eurasian_curlew_Liaoning_ZH-385_2014_H13N8	Asia	Shorebird
AKD00248_A_Eurasian_curlew_Liaoning_ZH-186_2014_H13N6	Asia	Shorebird
AKH14243_A_black-headed_gull_Netherlands_7_2009_H13N2	Europe	Gull
ALZ48194_A_glaucous-winged_gull_Southcentral_Alaska_11JR02474_2011_H13N6	North America	Gull
APC30335_A_black-headed_gull_Netherlands_33_2009_H13N2	Europe	Gull
APC30428_A_black-headed_gull_Netherlands_27_2009_H13N2	Europe	Gull
APC30500_A_black-headed_gull_Netherlands_100_2012_H13N6	Europe	Gull
APC30523_A_black-headed_gull_Netherlands_11_2014_H13N6	Europe	Gull
APC30630_A_black-headed_gull_Netherlands_21_2011_H13N8	Europe	Gull
APC30635_A_black-headed_gull_Netherlands_4_2010_H13N8	Europe	Gull
APC30717_A_black-headed_gull_Netherlands_1_2009_H13N2	Europe	Gull
APC30805_A_black-headed_gull_Netherlands_29_2009_H13N6	Europe	Gull
APC30962_A_black-headed_gull_Netherlands_20_2008_H13N8	Europe	Gull
APC31015_A_black-headed_gull_Netherlands_42_2012_H13N6	Europe	Gull
APC31217_A_black-headed_gull_Netherlands_35_2011_H13N8	Europe	Gull
APC31242_A_black-headed_gull_Netherlands_5_2014_H13N6	Europe	Gull
APC31616_A_black-headed_gull_Netherlands_2_2012_H13N6	Europe	Gull
APC31802_A_black-headed_gull_Netherlands_27_2014_H13N2	Europe	Gull
APC31839_A_black-headed_gull_Netherlands_55_2008_H13N8	Europe	Gull
APC31896_A_black-headed_gull_Netherlands_105_2012_H13N6	Europe	Gull
APC31938_A_black-headed_gull_Netherlands_10_2007_H13N6	Europe	Gull
APC32170_A_black-headed_gull_Netherlands_12_2010_H13N2	Europe	Gull
APC32229_A_black-headed_gull_Netherlands_81_2012_H13N6	Europe	Gull
APC32540_A_black-headed_gull_Netherlands_1_2010_H13N6	Europe	Gull
APC32688_A_black-headed_gull_Netherlands_9_2008_H13N8	Europe	Gull
APC32703_A_black-headed_gull_Netherlands_7_2013_H13N8	Europe	Gull
APC32983_A_black-headed_gull_Netherlands_26_2014_H13N6	Europe	Gull
APH09496_A_glaucous-winged_gull_Alaska_387_2013_H13N2	Europe	Gull
APH09591_A_glaucous-winged_gull_Alaska_545_2013_H13N2	Europe	Gull
APY16422_A_glaucous-winged_gull_Southcentral_Alaska_15MB01610_2015_H13N6	Europe	Gull
APY16562_A_glaucous-winged_gull_Southcentral_Alaska_15MB01557_2015_H13N6	Europe	Gull
APY16574_A_glaucous-winged_gull_Southcentral_Alaska_15MB01429_2015_H13N6	Europe	Gull
APY16674_A_glaucous-winged_gull_Southcentral_Alaska_15MB02016_2015_H13N8	Europe	Gull
ARX91482_A_black-headed_gull_Netherlands_97_2012_H13N6	Europe	Gull
ARX91696_A_black-headed_gull_Netherlands_25_2011_H13N8	Europe	Gull

ARX91926_A_black-headed_gull_Netherlands_74_2008_H13N8	Europe	Gull
ARX92299_A_black-headed_gull_Netherlands_8_2009_H13N2	Europe	Gull
ARX92583_A_black-headed_gull_Netherlands_50_2012_H13N6	Europe	Gull
ARX92851_A_black-headed_gull_Netherlands_1_2000_H13N8	Europe	Gull
ARX92920_A_black-headed_gull_Netherlands_38_2011_H13N8	Europe	Gull
ARX93396_A_black-headed_gull_Netherlands_55_2012_H13N6	Europe	Gull
ARX93594_A_black-headed_gull_Netherlands_138_2012_H13N6	Europe	Gull
ARX94378_A_black-headed_gull_Netherlands_120_2012_H13N6	Europe	Gull
ARX94432_A_black-headed_gull_Netherlands_88_2008_H13N8	Europe	Gull
ARX94508_A_black-headed_gull_Netherlands_64_2008_H13N8	Europe	Gull
ARX94613_A_black-headed_gull_Netherlands_37_2012_H13N6	Europe	Gull
ARX94626_A_black-headed_gull_Netherlands_18_2012_H13N6	Europe	Gull
ARX94839_A_yellow-legged_gull_Georgia_4_2012_H13N8	Europe	Gull
ARX94985_A_black-headed_gull_Netherlands_13_2014_H13N2	Europe	Gull
ARX95000_A_Mediterranean_gull_Netherlands_1_2012_H13N6	Europe	Gull
ARX95064_A_black-headed_gull_Netherlands_84_2012_H13N6	Europe	Gull
ARX95065_A_black-headed_gull_Netherlands_18_2008_H13N8	Europe	Gull
ASN77221_A_herring_gull_Massachusetts_A00080257_2006_H13N2	North America	Gull
ASO97744_A_black-tailed_gull_Weihai_115_2016_H13N2	Asia	Gull
AVO00172_A_laughing_gull_New_Jersey_UGAI17-2839_2017_H13N6	North America	Gull
AWF93296_A_environment_Chile_C7144_2016_H13N2	South America	Environment
AWV92286_A_gull_New_Jersey_UGAI15-3728_2015_H13N6	North America	Gull
AWV92298_A_gull_New_Jersey_UGAI15-3767_2015_H13N3	North America	Gull
AWW21182_A_environment_Valparaiso_P11_2017_H13N9	South America	Environment
AXB35128_A_environment_New_Jersey_AI00-2413_2000_H13N6	North America	Environment
AXE75376_A_environment_Chile_C20076_2016_H13N9	South America	Environment
AXF48995_A_American_oystercatcher_Chile_C20102_2016_H13N9	South America	Other Charadriiformes
AXF49018_A_Franklins_gull_Chile_C17421_2016_H13N9	South America	Gull
AXF49075_A_Franklins_gull_Chile_C20070_2016_H13N9	South America	Gull
AXF49116_A_Franklins_gull_Chile_C20118_2016_H13N9	South America	Gull
AXF49139_A_Franklins_gull_Chile_C20373_2016_H13N9	South America	Gull
AXF49349_A_environment_Chile_C20125_2016_H13N9	South America	Environment
AXF49608_A_kelp_gull_Chile_C27733_2017_H13N8	South America	Gull
AXF49618_A_kelp_gull_Chile_C8594_2016_H13N2	South America	Gull
AXO65323_A_ring-billed_gull_Minnesota_OPMNAI0816_2017_H13N8	North America	Gull
AXO65335_A_ring-billed_gull_Minnesota_CLMNAI1320_2017_H13N8	North America	Gull
AXO65347_A_ring-billed_gull_Minnesota_OPMNAI0926_2017_H13N6	North America	Gull
AXO65453_A_ring-billed_gull_Minnesota_CLMNAI1025_2017_H13N6	North America	Gull
AXO65524_A_ring-billed_gull_Minnesota_CLMNAI0891_2017_H13N6	North America	Gull
AXO65663_A_ring-billed_gull_Minnesota_CLMNAI1199_2017_H13N2	North America	Gull
AXO65887_A_ring-billed_gull_Minnesota_CLMNAI1501_2017_H13N2	North America	Gull
AXO65899_A_ring-billed_gull_Minnesota_CLMNAI1529_2017_H13N6	North America	Gull
AXO65911_A_ring-billed_gull_Minnesota_CLMNAI1539_2017_H13N6	North America	Gull
AZR38854_A_laughing_gull_Delaware_Bay_451_2018_H13N6	North America	Gull
AZV03327_A_laughing_gull_Delaware_Bay_458_2018_H13N6	North America	Gull
BAF37822_A_duck_Siberia_272_1998_H13N6	Asia	Anseriformes
BBB38801_A_duck_Hokkaido_W189_2006_H13N6	Asia	Anseriformes
CCH50639_A_common_gull_Norway_10_1313_2009_H13N2	Europe	Gull
QBL97178_A_red_knot_Delaware_Bay_489_2010_H13N6	North America	Shorebird
QBM14654_A_herring_gull_Delaware_Bay_22_2014_H13N6	North America	Gull
QDP69669_A_Glaucous-Winged_Gull_Southcentral_Alaska_17MB03606_2017_H13N2	North America	Gull
QDP69681_A_Glaucous-Winged_Gull_Southcentral_Alaska_17MB03625_2017_H13N2	North America	Gull
QDP69693_A_Glaucous-Winged_Gull_Southcentral_Alaska_17MB03638_2017_H13N2	North America	Gull
QDP69717_A_Glaucous-Winged_Gull_Southcentral_Alaska_18MB00667_2018_H13N6	North America	Gull
QGR25235_A_Red_Knot_Delaware_Bay_3_2019_H13N6	North America	Shorebird
QOL20603_A_slender-billed_gull_Ukraine_CHBZ-11_2009_H13N2	Europe	Gull
QTO33864_A_Red_Knot_Delaware_Bay_605_2020_H13N6	North America	Shorebird
QUD20219_A_Environmental_Santo_Domingo_GF89_2014_H13N2	South America	Environment
QUE37729_A_Glaucous-Winged_Gull_Alaska_20MB02182_2020_H13N6	North America	Gull
QUE37735_A_Glaucous-Winged_Gull_Alaska_20MB02831_2020_H13N6	North America	Gull
QUE37845_A_Glaucous-Winged_Gull_Alaska_20MB02534_2020_H13N6	North America	Gull
SKH13N6_NP	SKH13N6	Gull
UEE12804_A_Kelp_gull_Peru_M8_2019_H13N6	South America	Gull
UEF69691_A_silver_gull_Tasmania_06-0349-105_2006_H13N6	Oceania	Gull
UNA44619_A_Red_Knot_Delaware_224_2021_H13N6	North America	Shorebird
UZU88930_A_kelp_gull_Chile_C28937_2017_H13N8	South America	Gull
UZU88954_A_kelp_gull_Chile_C39002_2018_H13N2	South America	Gull
UZU89025_A_environment_Chile_C39659_2018_H13N2	South America	Environment
UZU89149_A_kelp_gull_Chile_C41225_2018_H13N6	South America	Gull
UZU89170_A_Franklins_gull_Chile_C42119_2018_H13N2	South America	Gull
UZU89226_A_kelp_gull_Chile_C41186_2018_H13N8	South America	Gull
UZU89273_A_environment_Chile_C39624_2018_H13N2	South America	Environment
UZU89320_A_southern_lapwing_Chile_C36480_2018_H13N8	South America	Other Charadriiformes
UZU89508_A_yellow-billed_teal_Chile_C38424_2018_H13N8	South America	Anseriformes
WCJ14705_A_Great_black-backed_gull_DE_844_2022_H13N2	North America	Gull
WCJ14787_A_Laughing_Gull_DE_801_2022_H13N2	North America	Gull
WEV00542_A_Franklins_gull_USA_D21-004347-2_MNAI1144_2017_H13N2	North America	Gull
WEV00543_A_Franklins_gull_USA_D21-004347-3_MNAI1172_2017_H13N6	North America	Gull
