# Supplementary material for: Genetic Characterization and Phylogeographic Analysis of the First H13N6 Avian Influenza Virus Isolated from Vega Gull in South Korea
Source: Viruses. 2024 Feb 12;16(2):285. doi: 10.3390/v16020285 (PMC10891532; doi:10.3390/v16020285)
Supplement: Supplementary file 1 [file viruses-16-00285-s001.zip › Table S7 NA isolates.pdf]

**Table S7.** List of isolates used in this study for NA gene analysis

| ISOLATE_ID | LOCATION | HOST |
|---|---|---|
| AAO62040_A_quail_Californa_1_1990_H4N6 | North America | Galliformes |
| AAO62049_A_common_murre_Alaska_305_1976_H1N6 | North America | Alcidae |
| AAO62050_A_pintail_duck_Alberta_712_1980_H3N6 | North America | Anseriformes |
| AAO62064_A_duck_New_Zealand_31_1976_H4N6 | Oceania | Anseriformes |
| ABB19110_A_pintail_Alberta_189_1982_H6N6 | North America | Anseriformes |
| ABB19439_A_mallard_duck_ALB_20_1976_H4N6 | North America | Anseriformes |
| ABB19510_A_mallard_Alberta_42_1977_H1N6 | North America | Anseriformes |
| ABB19663_A_blue-winged_teal_ALB_286_1977_H3N6 | North America | Anseriformes |
| ABB19777_A_pintail_duck_ALB_159_1977_H4N6 | North America | Anseriformes |
| ABB20119_A_herring_gull_Delaware_660_1988_H13N6 | North America | Gull |
| ABB20154_A_sanderling_Delaware_1258_1986_H6N6 | North America | Shorebird |
| ABB20257_A_duck_HK_231_1977_H3N6 | Asia | Anseriformes |
| ABB20327_A_duck_Hong_Kong_147_1977_H9N6 | Asia | Anseriformes |
| ABB20375_A_gray_teal_Australia_3_1979_H4N6 | Oceania | Anseriformes |
| ABB21764_A_gull_Minnesota_945_1980_H13N6 | North America | Gull |
| ABB87413_A_pintail_duck_ALB_462_1979_H3N6 | North America | Anseriformes |
| ABB87476_A_canvasback_duck_ALB_274_1977_H4N6 | North America | Anseriformes |
| ABB87498_A_redhead_duck_ALB_74_1977_H4N6 | North America | Anseriformes |
| ABB87509_A_blue-winged_teal_ALB_243_1977_H4N6 | North America | Anseriformes |
| ABB87607_A_ruddy_turnstone_New_Jersey_47_1985_H4N6 | North America | Shorebird |
| ABB87670_A_blue-winged_teal_ALB_293_1994_H4N6 | North America | Anseriformes |
| ABB87992_A_pintail_duck_ALB_584_1984_H10N6 | North America | Anseriformes |
| ABB88270_A_duck_Hong_Kong_365_1978_H4N6 | Asia | Anseriformes |
| ABB88351_A_duck_Potsdam_2216-4_1984_H5N6 | Europe | Anseriformes |
| ABB90177_A_redhead_duck_ALB_116_1985_H4N6 | North America | Anseriformes |
| ABI84666_A_turkey_Minnesota_957_1980_H6N6 | North America | Galliformes |
| ABI84809_A_mallard_duck_New_York_157_1986_H3N6 | North America | Anseriformes |
| ABI85203_A_shorebird_Delaware_224_1997_H13N6 | North America | Shorebird |
| ABO52503_A_mallard_Ohio_178_1990_H4N6 | North America | Anseriformes |
| ACA04737_A_duck_England_1_1956_H11N6 | Europe | Anseriformes |
| ACA14293_A_duck_Korea_LPM38_2005_H3N6 | Asia | Anseriformes |
| ACE78976_A_black-headed_gull_Astrakhan_44_1988_H13N6 | Europe | Gull |
| ACE78977_A_Larus_argentatus_Astrakhan_458_1985_H13N6 | Europe | Gull |
| ACF25168_A_pintail_Minnesota_479_1999_H3N6 | North America | Anseriformes |
| ACF25179_A_mallard_MN_515_2000_H3N6 | North America | Anseriformes |
| ACV41595_A_mallard_New_Zealand_1615-17_2004_H4N6 | Oceania | Anseriformes |
| ACV41672_A_mallard_Interior_Alaska_7MP1050R1_2007_H4N6 | North America | Anseriformes |
| ACV86833_A_black_headed_gull_Mongolia_1766_2006_H13N6 | Asia | Gull |
| ACV86843_A_Mongolian_gull_Mongolia_401_2007_H13N6 | Asia | Gull |
| ACV86853_A_Mongolian_gull_Mongolia_405_2007_H13N6 | Asia | Gull |
| ACX55392_A_green-winged_teal_Nova_Scotia_14687_2005_H4N6 | North America | Anseriformes |
| ADD11586_A_Muscovy_duck_Fujian_FZ01_2008_H6N6 | Asia | Anseriformes |
| ADD92021_A_great_black-headed_gull_Atyrau_743_2004_H13N6 | Asia | Gull |
| ADD92022_A_great_black-headed_gull_Atyrau_744_2004_H13N6 | Asia | Gull |
| ADD92023_A_great_black-headed_gull_Atyrau_767_2004_H13N6 | Asia | Gull |
| ADD92024_A_great_black-headed_gull_Atyrau_773_2004_H13N6 | Asia | Gull |
| ADE75159_A_mallard_Sweden_41_2002_H10N6 | Europe | Anseriformes |
| ADF45448_A_duck_Victoria_512_2007_H7N6 | Oceania | Anseriformes |
| ADH96329_A_blue-winged_teal_Texas_Sg-00076_2007_H4N6 | North America | Anseriformes |
| ADQ20587_A_mallard_Netherlands_4_2006_H10N6 | Europe | Anseriformes |
| ADR00749_A_mallard_Interior_Alaska_7MP2230_2007_H1N6 | North America | Anseriformes |
| ADT79262_A_blue-winged_teal_Texas_Sg-00083_2007_H3N6 | North America | Anseriformes |
| ADT79637_A_blue-winged_teal_Texas_Sg-00206_2007_H4N6 | North America | Anseriformes |
| AEA04433_A_duck_United_Kingdom_1_1956_H11N6 | Europe | Anseriformes |
| AEB40996_A_mallard_Ohio_42_1989_H10N6 | North America | Anseriformes |
| AEB66908_A_gull_Maryland_704_1977_H13N6 | North America | Gull |
| AEB66919_A_duck_Wisconsin_480_1979_H12N6 | North America | Anseriformes |
| AEF59115_A_duck_Eastern_China_8_2009_H6N6 | Asia | Anseriformes |
| AEF59117_A_duck_Eastern_China_10_2009_H6N6 | Asia | Anseriformes |
| AEK50178_A_mallard_California_10064_2008_H1N6 | North America | Anseriformes |
| AET74364_A_blue-winged_teal_Alberta_134_1989_H3N6 | North America | Anseriformes |
| AET74398_A_ruddy_turnstone_New_Jersey_138_1990_H3N6 | North America | Shorebird |
| AET74414_A_semi-palmated_sandpiper_New_Jersey_237_1990_H3N6 | North America | Shorebird |
| AET74513_A_mallard_Alberta_72_1994_H3N6 | North America | Anseriformes |
| AET75150_A_semi-palmated_sandpiper_New_Jersey_253_1990_H3N6 | North America | Shorebird |
| AET77279_A_shorebird_Delaware_Bay_140_2005_H3N6 | North America | Shorebird |
| AET77430_A_shorebird_Delaware_Bay_518_2005_H3N6 | North America | Shorebird |
| AFF25709_A_duck_Shantou_1165_2006_H6N6 | Asia | Anseriformes |
| AFF25720_A_wild_duck_Shantou_2441_2006_H6N6 | Asia | Anseriformes |
| AFF26072_A_duck_Guangxi_2281_2007_H6N6 | Asia | Anseriformes |
| AFF26484_A_duck_Hunan_1668_2007_H6N6 | Asia | Anseriformes |
| AFF26544_A_duck_Shantou_12958_2006_H6N6 | Asia | Anseriformes |
| AFF27141_A_duck_Guizhou_1128_2007_H6N6 | Asia | Anseriformes |
| AFO83149_A_duck_Thailand_CU-LM7291C_2010_H7N6 | Asia | Anseriformes |
| AFP20873_A_ring-billed_gull_Quebec_02434-1_2009_H13N6 | North America | Gull |
| AFQ60311_A_wild_duck_Korea_PSC6-1_2009_H4N6 | Asia | Anseriformes |
| AFQ60333_A_wild_bird_Korea_GD42_2005_H4N6 | Asia | Wildbird |
| AFQ60344_A_duck_Korea_DY104_2007_H4N6 | Asia | Anseriformes |

| | | |
|---|---|---|
| AFQ60376_A_wild_duck_Korea_CSM4-28_2010_H4N6 | Asia | Anseriformes |
| AFV80689_A_mallard_California_1154_2010_H4N6 | North America | Anseriformes |
| AGB98186_A_glaucous-winged_gull_Southeastern_Alaska_9JR0822R0_2009_H13N6 | North America | Gull |
| AGC73416_A_mallard_Ohio_11OS2049_2011_H3N6 | North America | Anseriformes |
| AGG84038_A_American_black_duck_New_Brunswick_00971_2010_H10N6 | North America | Anseriformes |
| AGG84129_A_American_black_duck_New_Brunswick_00998_2010_H12N6 | North America | Anseriformes |
| AGK43189_A_blue-winged_teal_Louisiana_AI09-4180_2009_H4N6 | North America | Anseriformes |
| AGL57478_A_herring_gull_New_Jersey_AI09-1105_2009_H13N6 | North America | Gull |
| AGL58139_A_ruddy_turnstone_New_Jersey_AI05-1058_2005_H11N6 | North America | Shorebird |
| AGL58199_A_ruddy_turnstone_New_Jersey_1321394_2005_H3N6 | North America | Shorebird |
| AGL59905_A_blue-winged_teal_Minnesota_Sg-00899_2008_H10N6 | North America | Anseriformes |
| AGL60137_A_ruddy_turnstone_New_Jersey_AI09-294_2009_H13N6 | North America | Shorebird |
| AGQ81863_A_wild_waterfowl_Hong_Kong_MPM3375_2011_H7N6 | Asia | Anseriformes |
| AGY42192_A_mallard_Netherlands_5_2001_H3N6 | Europe | Anseriformes |
| AHM99113_A_thick-billed_murre_Alaska_167_1976_H1N6 | North America | Alcidae |
| AHM99125_A_common_murre_Alaska_186_1976_H1N6 | North America | Alcidae |
| AHM99137_A_thick-billed_murre_Alaska_191_1976_H1N6 | North America | Alcidae |
| AHM99485_A_blue-winged_teal_Wisconsin_376_1983_H4N6 | North America | Anseriformes |
| AHM99496_A_mallard_Wisconsin_408_1983_H3N6 | North America | Anseriformes |
| AHN00312_A_duck_Wisconsin_2731_1985_H4N6 | North America | Anseriformes |
| AHN00348_A_duck_Wisconsin_2810_1985_H3N6 | North America | Anseriformes |
| AHN00420_A_mallard_Wisconsin_1944_1983_H4N6 | North America | Anseriformes |
| AHN00492_A_mallard_Saskatchewan_117_1982_H4N6 | North America | Anseriformes |
| AHN00576_A_blue-winged_teal_Wisconsin_393_1984_H4N6 | North America | Anseriformes |
| AHN00887_A_blue-winged_teal_Wisconsin_402_1983_H4N6 | North America | Anseriformes |
| AHN01190_A_snipe_Wisconsin_330_1976_H4N6 | North America | Shorebird |
| AHN01251_A_thick-billed_murre_Alaska_170_1976_H1N6 | North America | Alcidae |
| AHN01371_A_thick-billed_murre_Alaska_192_1976_H1N6 | North America | Alcidae |
| AHN01419_A_thick-billed_murre_Alaska_335_1976_H1N6 | North America | Alcidae |
| AHN01455_A_common_murre_Alaska_434_1976_H1N6 | North America | Alcidae |
| AHN01707_A_mallard_Wisconsin_607_1977_H4N6 | North America | Anseriformes |
| AHN02077_A_thick-billed_murre_Alaska_206_1976_H1N6 | North America | Alcidae |
| AHN02113_A_thick-billed_murre_Alaska_210_1976_H1N6 | North America | Alcidae |
| AHN02221_A_common_murre_Alaska_312_1976_H1N6 | North America | Alcidae |
| AHN02293_A_mallard_Wisconsin_18_1977_H3N6 | North America | Anseriformes |
| AHN02353_A_blue-winged_teal_Wisconsin_278_1975_H4N6 | North America | Anseriformes |
| AHN03282_A_mallard_Wisconsin_364_1984_H4N6 | North America | Anseriformes |
| AHN03402_A_mallard_Wisconsin_418_1976_H3N6 | North America | Anseriformes |
| AHN03524_A_duck_Minnesota_A_1975_H4N6 | North America | Anseriformes |
| AHN03872_A_mallard_Wisconsin_503_1976_H12N6 | North America | Anseriformes |
| AHN04146_A_green-winged_teal_Wisconsin_121_1980_H6N6 | North America | Anseriformes |
| AHN04575_A_blue-winged_teal_Wisconsin_19_1974_H4N6 | North America | Anseriformes |
| AHN04765_A_mallard_Wisconsin_14_1974_H4N6 | North America | Anseriformes |
| AHN04789_A_blue-winged_teal_Wisconsin_123_1978_H3N6 | North America | Anseriformes |
| AHN13651_A_duck_Thailand_CU-12658T_2012_H11N6 | Asia | Anseriformes |
| AHZ13549_A_mallard_New_Zealand_223-31_2005_H4N6 | Oceania | Anseriformes |
| AHZ13556_A_mallard_New_Zealand_527-11_2005_H4N6 | Oceania | Anseriformes |
| AHZ13561_A_mallard_New_Zealand_449-66_2004_H4N6 | Oceania | Anseriformes |
| AHZ37858_A_mallard_Sweden_93475_2009_H10N6 | Europe | Anseriformes |
| AHZ39228_A_black-headed_gull_Republic_of_Georgia_7_2011_H13N6 | Europe | Gull |
| AHZ39240_A_yellow-legged_gull_Georgia_1_2011_H13N6 | Europe | Gull |
| AHZ39383_A_black-headed_gull_Republic_of_Georgia_2_2012_H13N6 | Europe | Gull |
| AHZ39442_A_yellow-legged_gull_Republic_of_Georgia_2_2012_H13N6 | Europe | Gull |
| AHZ39454_A_yellow-legged_gull_Republic_of_Georgia_3_2012_H13N6 | Europe | Gull |
| AIA61948_A_environment_Korea_UPO218_2008_H1N6 | Asia | Environment |
| AIN76265_A_herring_gull_Maine_ND0000047_2013_H13N6 | North America | Gull |
| AIR93945_A_chicken_Laos_LPQ001_2014_H5N6 | Asia | Galliformes |
| AJD11429_A_chicken_Jiangxi_13200_2014_H10N6 | Asia | Galliformes |
| AJD11631_A_chicken_Shenzhen_1061_2013_H5N6 | Asia | Galliformes |
| AJD14189_A_migratory_duck_Jiangxi_10974_2005_H10N6 | Asia | Anseriformes |
| AJS16193_A_goose_Eastern_China_S0513_2013_H5N6 | Asia | Anseriformes |
| AKF34926_A_mallard_Maryland_09OS2237_2009_H10N6 | North America | Anseriformes |
| AKF35048_A_environment_Maryland_12OS1465_2012_H3N6 | North America | Environment |
| ALZ46750_A_ring-billed_gull_Massachusetts_12DC00060_2012_H13N6 | North America | Gull |
| ALZ46774_A_gull_Massachusetts_12JR00671_2012_H13N6 | North America | Gull |
| ALZ47461_A_mallard_Interior_Alaska_11BM02987_2011_H2N6 | North America | Anseriformes |
| AMQ31640_A_surf_scoter_Maryland_14OS1407_2014_H10N6 | North America | Anseriformes |
| APY16561_A_glaucous-winged_gull_Southcentral_Alaska_15MB01557_2015_H13N6 | North America | Gull |
| ARI67198_A_mallard_California_UCD353_2014_H2N6 | North America | Anseriformes |
| BAF37823_A_duck_Siberia_272_1998_H13N6 | Asia | Anseriformes |
| BAF46903_A_duck_Hong_Kong_22B_1976_H3N6 | Asia | Anseriformes |
| BAF46907_A_gull_Maryland_704_1977_H13N6 | North America | Gull |
| BAJ10562_A_duck_Vietnam_OIE-2334_2010_H9N6 | Asia | Anseriformes |
| BAN14681_A_duck_Vietnam_LBM235_2012_H3N6 | Asia | Anseriformes |
| BAP90406_A_duck_Vietnam_LBM360c1-4-1_2013_H5N6 | Asia | Anseriformes |
| BAQ35850_A_duck_Vietnam_LBM439_2013_H6N6 | Asia | Anseriformes |
| BAR64286_A_duck_Vietnam_HU1-1152_2014_H5N6 | Asia | Anseriformes |
| BAR71099_A_muscovy_duck_Quang_Ninh_4c111_2013_H5N6 | Asia | Anseriformes |
| BAW94780_A_duck_Japan_AQ-HE72_2015_H5N6 | Asia | Anseriformes |
| SKH13N6_NA | SKH13N6 | Gull |
