# Supplementary material for: Genetic Characterization and Phylogeographic Analysis of the First H13N6 Avian Influenza Virus Isolated from Vega Gull in South Korea
Source: Viruses. 2024 Feb 12;16(2):285. doi: 10.3390/v16020285 (PMC10891532; doi:10.3390/v16020285)
Supplement: Supplementary file 1 [file viruses-16-00285-s001.zip › Table S8 M isolates.pdf]

Table S8. List of isolates used in this study for M gene analysis

ISOLATE_ID	LOCATION	HOST
AB284991_A_duck_Siberia_272_1998_H13N6	Asia	Anseriformes
CY003895_A_herring_gull_DE_475_1986_H13N2	North America	Gull
CY004444_A_shorebird_DE_68_2004_H13N9	North America	Shorebird
CY005065_A_laughing_gull_Delaware_Bay_2838_1987__H13N2	North America	Gull
CY014800_A_turkey_Minnesota_1012_1990__H13N2	North America	Galliformes
CY015147_A_shorebird_Delaware_224_1997__H13N6	North America	Shorebird
CY077001_A_black-headed_gull_Sweden_1_2005_H13N8	Europe	Gull
CY089638_A_gull_Maryland_704_1977__H13N6	North America	Gull
CY126289_A_ruddy_turnstone_Delaware_Bay_520_1988__H13N9	North America	Shorebird
CY185364_A_common_snipe_Republic_of_Georgia_1_2012_H13N2	Europe	Shorebird
CY185490_A_black-headed_gull_Republic_of_Georgia_7_2011_H13N6	Europe	Gull
CY185514_A_black-headed_gull_Georgia_1_2011_H13N8	Europe	Gull
CY195648_A_ring-billed_gull_Massachusetts_12DC00060_2012_H13N6	North America	Gull
CY195664_A_gull_Massachusetts_12JR00671_2012_H13N6	North America	Gull
CY195802_A_ring-billed_gull_Massachusetts_13DC30736-1_2013_H13N8	North America	Gull
CY196612_A_glaucous-winged_gull_Southcentral_Alaska_11JR02474_2011_H13N6	North America	Gull
CY213621_A_glaucous-winged_gull_Southcentral_Alaska_15MB01557_2015_H13N6	North America	Gull
CY213696_A_glaucous-winged_gull_Southcentral_Alaska_15MB02016_2015_H13N8	North America	Gull
EU580589_A_black-headed_gull_Astrakhan_65_1983__H13N6	Europe	Gull
EU580591_A_gull_Astrakhan_1846_1998_H13N6	Europe	Gull
GQ907303_A_black_headed_gull_Mongolia_1766_2006_H13N6	Asia	Gull
GQ907319_A_Mongolian_gull_Mongolia_405_2007_H13N6	Asia	Gull
GU052229_A_gull_Astrakhan_1846_1998_H13N6	Europe	Gull
GU052234_A_blackhead_gull_Astrakhan_1421_1979__H13N6	Europe	Gull
GU052240_A_black-headed_gull_Astrakhan_458_1985__H13N6	Europe	Gull
GU052246_A_black-headed_gull_Astrakhan_44_1988__H13N6	Europe	Gull
HM060045_A_glaucous_gull_Alaska_44199-006_2006_H13N9	North America	Gull
HM060047_A_lesser_snow_goose_Alaska_44199-115_2006_H13N9	North America	Anseriformes
JF775473_A_herring_gull_Mongolia_454_2008_H13N8	Asia	Gull
JX982948_A_gull_Maryland_1824_1978__H13N9	North America	Gull
KF462335_A_common_gull_Altai_811_2011_H13N2	Asia	Gull
KF772959_A_seagull_Chile_5775_2009_H13N9	South America	Gull
KJ372723_A_whale_Maine_328B_1984__H13N2	North America	Mammal
KM374021_A_herring_gull_Maine_ND0000047_2013_H13N6	North America	Gull
KR010438_A_Eurasian_curlew_Liaoning_ZH-186_2014_H13N6	Asia	Shorebird
KR010446_A_Eurasian_curlew_Liaoning_ZH-385_2014_H13N8	Asia	Shorebird
KR087567_A_black-headed_gull_Netherlands_7_2009_H13N2	Europe	Gull
KX977643_A_black-headed_gull_Netherlands_10_2014_H13N6	Europe	Gull
KX977796_A_black-headed_gull_Netherlands_21_2008_H13N8	Europe	Gull
KX977809_A_black-headed_gull_Netherlands_20_2008_H13N8	Europe	Gull
KX978014_A_black-headed_gull_Netherlands_10_2010_H13N8	Europe	Gull
KX978042_A_black-headed_gull_Netherlands_15_2011_H13N8	Europe	Gull
KX978078_A_black-headed_gull_Netherlands_4_2010_H13N8	Europe	Gull
KX978087_A_black-headed_gull_Netherlands_9_2008_H13N8	Europe	Gull
KX978210_A_black-headed_gull_Netherlands_7_2013_H13N8	Europe	Gull
KX978395_A_black-headed_gull_Netherlands_25_2014_H13N2	Europe	Gull
KX978443_A_black-headed_gull_Netherlands_2_2011_H13N8	Europe	Gull
KX979037_A_black-headed_gull_Netherlands_39_2009_H13N6	Europe	Gull
KX979201_A_black-headed_gull_Netherlands_1_2010_H13N6	Europe	Gull
KX979210_A_black-headed_gull_Netherlands_83_2008_H13N8	Europe	Gull
KY131020_A_glaucous-winged_gull_Alaska_387_2013_H13N2	North America	Gull
KY131075_A_glaucous-winged_gull_Alaska_544_2013_H13N2	North America	Gull
KY131083_A_glaucous-winged_gull_Alaska_545_2013_H13N2	North America	Gull
LC339630_A_duck_Hokkaido_W189_2006_H13N6	Asia	Anseriformes
M63530_A_gull_Maryland_1815_1979__H13N6	North America	Gull
M63539_A_gull_Maryland_1824_1978__H13N9	North America	Gull
MF145905_A_black-headed_gull_Netherlands_9_2013_H13N8	Europe	Gull
MF145975_A_black-headed_gull_Netherlands_1_2000_H13N8	Europe	Gull
MF146096_A_black-headed_gull_Netherlands_30_2008_H13N8	Europe	Gull
MF146491_A_black-headed_gull_Netherlands_16_2014_H13N6	Europe	Gull
MF146532_A_yellow-legged_gull_Georgia_4_2012_H13N8	Europe	Gull
MF146669_A_black-headed_gull_Netherlands_95_2012_H13N6	Europe	Gull
MF147149_A_black-headed_gull_Netherlands_49_2012_H13N6	Europe	Gull
MF461183_A_black-tailed_gull_Weihai_115_2016_H13N2	Asia	Gull
MH134621_A_brown-hooded_gull_Chile_C10765_2016_H13N2	South America	Gull
MH498651_A_environment_Chile_C20128_2016_H13N9	South America	Environment
MH498975_A_Franklins_gull_Chile_C20069_2016_H13N9	South America	Gull
MH499031_A_Franklins_gull_Chile_C20118_2016_H13N9	South America	Gull
MH499169_A_Franklins_gull_Chile_C17421_2016_H13N9	South America	Gull
MH499209_A_black_skimmer_Chile_C20108_2016_H13N9	South America	Shorebird
MH501031_A_gull_New_Jersey_UGAI15-3416_2015_H13N6	North America	Gull
MH501055_A_gull_New_Jersey_UGAI15-3767_2015_H13N3	North America	Gull
MH578852_A_semipalmated_sandpiper_Delaware_Bay_142_2007_H13N9	North America	Shorebird
MH579027_A_kelp_gull_Delaware_Bay_304_2007_H13N9	North America	Gull
MH733687_A_ruddy_turnstone_Delaware_Bay_204_2009_H13N6	North America	Shorebird
MH763876_A_ring-billed_gull_Minnesota_OPMNAI0926_2017_H13N6	North America	Gull
MH763995_A_ring-billed_gull_Minnesota_CLMNAI0891_2017_H13N6	North America	Gull
MH764222_A_ring-billed_gull_Minnesota_CLMNAI1301_2017_H13N2	North America	Gull

MH764254_A_ring-billed_gull_Minnesota_CLMNAI1539_2017_H13N6	North America	Gull
MK637083_A_herring_gull_Delaware_Bay_22_2014_H13N6	North America	Gull
MN210164_A_Glaucous-Winged_Gull_Southcentral_Alaska_17MB03606_2017_H13N2	North America	Gull
MN210180_A_Glaucous-Winged_Gull_Southcentral_Alaska_17MB03638_2017_H13N2	North America	Gull
MN210196_A_Glaucous-Winged_Gull_Southcentral_Alaska_18MB00667_2018_H13N6	North America	Gull
MN210213_A_Glaucous-Winged_Gull_Southcentral_Alaska_18MB00688_2018_H13N6	North America	Gull
MW126757_A_slender-billed_gull_Ukraine_CHBZ-11_2009_H13N2	Europe	Gull
MZ005192_A_Environmental_Santo_Domingo_GF89_2014_H13N2	South America	Environment
MZ015622_A_Glaucous-Winged_Gull_Alaska_20MB02182_2020_H13N6	North America	Gull
MZ015717_A_Glaucous-Winged_Gull_Alaska_20MB02820_2020_H13N6	North America	Gull
OL355046_A_Kelp_gull_Peru_M8_2019_H13N6	South America	Gull
OL369951_A_silver_gull_Tasmania_06-0349-105_2006_H13N6	Oceania	Gull
OM965828_A_Red_Knot_Delaware_224_2021_H13N6	North America	Shorebird
OP888638_A_environment_Chile_C39659_2018_H13N2	South America	Environment
OP888736_A_Franklins_gull_Chile_C42119_2018_H13N2	South America	Gull
OP888963_A_yellow-billed_teal_Chile_C38424_2018_H13N8	South America	Anseriformes
OQ366633_A_Great_black-backed_gull_DE_844_2022_H13N2	North America	Gull
OQ366761_A_Laughing_Gull_DE_827_2022_H13N2	North America	Gull
OQ691621_A_Franklins_gull_USA_D21-004347-2_MNAI1144_2017_H13N2	North America	Gull
OQ691622_A_Franklins_gull_USA_D21-004347-3_MNAI1172_2017_H13N6	North America	Gull
SKH13N6_M	SKH13N6	Gull
