# Supplementary material for: Genetic Characterization and Phylogeographic Analysis of the First H13N6 Avian Influenza Virus Isolated from Vega Gull in South Korea
Source: Viruses. 2024 Feb 12;16(2):285. doi: 10.3390/v16020285 (PMC10891532; doi:10.3390/v16020285)
Supplement: Supplementary file 1 [file viruses-16-00285-s001.zip › Table S9 NS isolates.pdf]

Table S9. List of isolates used in this study for NS gene analysis

ISOLATE_ID	LOCATION	HOST
AB285090_A_duck_Siberia_272_1998_H13N6	Asia	Anseriformes
CY003898_A_herring_gull_DE_475_1986_H13N2	North America	Gull
CY004454_A_herring_gull_NJ_782_1986_H13N2	North America	Gull
CY005373_A_shorebird_NJ_840_1986_H13N3	North America	Shorebird
CY005384_A_laughing_gull_DE_554_1988_H13N3	North America	Gull
CY014802_A_turkey_Minnesota_1012_1990_H13N2	North America	Galliformes
CY043892_A_shorebird_Delaware_221_2006_H13N9	North America	Shorebird
CY077004_A_black-headed_gull_Sweden_1_2005_H13N8	Europe	Gull
CY089641_A_gull_Maryland_704_1977_H13N6	North America	Gull
CY094907_A_silver_gull_Tasmania_062_2006_H13N6	Oceania	Gull
CY125313_A_hooded_merganser_New_Brunswick_03750_2009_H13N6	North America	Anseriformes
CY126292_A_ruddy_turnstone_Delaware_Bay_520_1988_H13N9	North America	Shorebird
CY128962_A_mallard-black_duck_hybrid_New_Brunswick_03736_2009_H13N6	North America	Anseriformes
CY130344_A_glaucous-winged_gull_Southeastern_Alaska_9JR0822R0_2009_H13N6	North America	Gull
CY140257_A_laughing_gull_New_Jersey_Sg-00568_2008_H13N9	North America	Gull
CY144206_A_ring-billed_gull_Georgia_AI00-2658_2000_H13N6	North America	Gull
CY144985_A_laughing_gull_New_Jersey_Sg-00485_2008_H13N9	North America	Gull
CY145943_A_ruddy_turnstone_New_Jersey_AI09-294_2009_H13N6	North America	Shorebird
CY184492_A_black-headed_gull_Sweden_74342_2008_H13N8	Europe	Gull
CY185359_A_Armenian_gull_Republic_of_Georgia_1_2012_H13N6	Europe	Gull
CY185367_A_common_snipe_Republic_of_Georgia_1_2012_H13N2	Europe	Shorebird
CY185493_A_black-headed_gull_Republic_of_Georgia_7_2011_H13N6	Europe	Gull
CY185501_A_yellow-legged_gull_Georgia_1_2011_H13N6	Europe	Gull
CY185517_A_black-headed_gull_Georgia_1_2011_H13N8	Europe	Gull
CY185573_A_black-headed_gull_Republic_of_Georgia_2_2012_H13N6	Europe	Gull
CY185605_A_yellow-legged_gull_Republic_of_Georgia_2_2012_H13N6	Europe	Gull
CY185613_A_yellow-legged_gull_Republic_of_Georgia_3_2012_H13N6	Europe	Gull
CY185661_A_black-headed_gull_Georgia_9_2011_H13N8	Europe	Gull
CY185669_A_yellow-legged_gull_Republic_of_Georgia_6_2012_H13N6	Europe	Gull
CY195651_A_ring-billed_gull_Massachusetts_12DC00060_2012_H13N6	North America	Gull
CY196615_A_glaucous-winged_gull_Southcentral_Alaska_11JR02474_2011_H13N6	North America	Gull
CY213531_A_glaucous-winged_gull_Southcentral_Alaska_15MB01610_2015_H13N6	North America	Gull
CY213624_A_glaucous-winged_gull_Southcentral_Alaska_15MB01557_2015_H13N6	North America	Gull
CY213632_A_glaucous-winged_gull_Southcentral_Alaska_15MB01429_2015_H13N6	North America	Gull
CY239284_A_herring_gull_Massachusetts_A00080257_2006_H13N2	North America	Gull
EU523139_A_kelp_gull_Argentina_LDC4_2006_H13N9	South America	Gull
EU580579_A_black-headed_gull_Astrakhan_458_1985_H13N6	Europe	Gull
GQ907314_A_Mongolian_gull_Mongolia_401_2007_H13N6	Asia	Gull
GU052231_A_gull_Astrakhan_1846_1998_H13N6	Europe	Gull
HM060070_A_lesser_snow_goose_Alaska_44199-115_2006_H13N9	North America	Anseriformes
HM060072_A_glaucous_gull_Alaska_44199-104_2006_H13N9	North America	Gull
JF775472_A_herring_gull_Mongolia_454_2008_H13N8	Asia	Gull
JX030402_A_mallard_Korea_SH38-45_2010_H13N2	Asia	Anseriformes
KC845078_A_herring_gull_Newfoundland_GR578_2011_H13N6	North America	Gull
KC845102_A_herring_gull_Newfoundland_GR657_2011_H13N6	North America	Gull
KF772960_A_seagull_Chile_5775_2009_H13N9	South America	Gull
KJ907715_A_mallard_Dalian_DZ-137_2013_H13N6	Asia	Anseriformes
KM374022_A_herring_gull_Maine_ND0000047_2013_H13N6	North America	Gull
KR010439_A_Eurasian_curlew_Liaoning_ZH-186_2014_H13N6	Asia	Shorebird
KR010447_A_Eurasian_curlew_Liaoning_ZH-385_2014_H13N8	Asia	Shorebird
KR087568_A_black-headed_gull_Netherlands_7_2009_H13N2	Europe	Gull
KR824562_A_gull_Peru_CH02_2009_H13N2	South America	Gull
KR824667_A_whimbrel_Peru_P41_2007_H13N2	South America	Shorebird
KX977674_A_black-headed_gull_Netherlands_96_2008_H13N8	Europe	Gull
KX977710_A_black-headed_gull_Netherlands_9_2009_H13N2	Europe	Gull
KX977864_A_black-headed_gull_Netherlands_83_2012_H13N6	Europe	Gull
KX977873_A_black-headed_gull_Netherlands_33_2009_H13N2	Europe	Gull
KX977931_A_black-headed_gull_Netherlands_21_2011_H13N8	Europe	Gull
KX977946_A_black-headed_gull_Netherlands_25_2008_H13N8	Europe	Gull
KX977976_A_black-headed_gull_Netherlands_12_2010_H13N2	Europe	Gull
KX978031_A_black-headed_gull_Netherlands_10_2013_H13N8	Europe	Gull
KX978109_A_black-headed_gull_Netherlands_21_2008_H13N8	Europe	Gull
KX978135_A_black-headed_gull_Netherlands_106_2012_H13N6	Europe	Gull
KX978139_A_black-headed_gull_Netherlands_1_2009_H13N2	Europe	Gull
KX978203_A_black-headed_gull_Netherlands_20_2008_H13N8	Europe	Gull
KX978219_A_black-headed_gull_Netherlands_4_2007_H13N6	Europe	Gull
KX978324_A_black-headed_gull_Netherlands_69_2012_H13N6	Europe	Gull
KX978474_A_black-headed_gull_Netherlands_55_2008_H13N8	Europe	Gull
KX978517_A_black-headed_gull_Netherlands_5_2014_H13N6	Europe	Gull
KX978518_A_black-headed_gull_Netherlands_8_2010_H13N2	Europe	Gull
KX978520_A_black-headed_gull_Netherlands_2_2012_H13N6	Europe	Gull
KX978597_A_black-headed_gull_Netherlands_1_2010_H13N6	Europe	Gull
KX978708_A_black-headed_gull_Netherlands_18_2009_H13N2	Europe	Gull
KX978741_A_black-headed_gull_Netherlands_72_2012_H13N6	Europe	Gull
KX978938_A_black-headed_gull_Netherlands_29_2009_H13N6	Europe	Gull
KX978962_A_black-headed_gull_Netherlands_31_2009_H13N2	Europe	Gull
KX979074_A_black-headed_gull_Netherlands_15_2014_H13N6	Europe	Gull
KX979221_A_black-headed_gull_Netherlands_10_2010_H13N8	Europe	Gull

KX979252_A_black-headed_gull_Netherlands_39_2009_H13N6	Europe	Gull
KX979254_A_black-headed_gull_Netherlands_25_2014_H13N2	Europe	Gull
KX979269_A_black-headed_gull_Netherlands_33_2012_H13N6	Europe	Gull
KX979518_A_black-headed_gull_Netherlands_13_2010_H13N2	Europe	Gull
KX979538_A_black-headed_gull_Netherlands_10_2014_H13N6	Europe	Gull
KX979567_A_black-headed_gull_Netherlands_6_2010_H13N8	Europe	Gull
KX979574_A_black-headed_gull_Netherlands_4_2010_H13N8	Europe	Gull
KY131021_A_glaucous-winged_gull_Alaska_387_2013_H13N2	North America	Gull
KY131076_A_glaucous-winged_gull_Alaska_544_2013_H13N2	North America	Gull
KY131084_A_glaucous-winged_gull_Alaska_545_2013_H13N2	North America	Gull
KY243233_A_black-headed_gull_Netherlands_23_2014_H13N2	Europe	Gull
LC339631_A_duck_Hokkaido_W189_2006_H13N6	Asia	Anseriformes
M80952_A_whale_Maine_328_1984_H13N2	North America	Mammal
M80958_A_gull_Minnesota_1352_1981_H13N6	North America	Gull
MF145958_A_black-headed_gull_Netherlands_75_2012_H13N6	Europe	Gull
MF145976_A_black-headed_gull_Netherlands_8_2009_H13N2	Europe	Gull
MF146105_A_black-headed_gull_Netherlands_2_2009_H13N2	Europe	Gull
MF146194_A_yellow-legged_gull_Georgia_4_2012_H13N8	Europe	Gull
MF146370_A_black-headed_gull_Netherlands_1_2000_H13N8	Europe	Gull
MF146440_A_black-headed_gull_Netherlands_38_2009_H13N2	Europe	Gull
MF146527_A_black-headed_gull_Netherlands_51_2012_H13N6	Europe	Gull
MF146704_A_black-headed_gull_Netherlands_14_2012_H13N6	Europe	Gull
MF146801_A_black-headed_gull_Netherlands_6_2009_H13N2	Europe	Gull
MF147077_A_black-headed_gull_Netherlands_38_2011_H13N8	Europe	Gull
MF147361_A_black-headed_gull_Netherlands_2_2010_H13N8	Europe	Gull
MF147986_A_black-headed_gull_Netherlands_122_2012_H13N6	Europe	Gull
MF148137_A_black-headed_gull_Netherlands_16_2014_H13N6	Europe	Gull
MF461184_A_black-tailed_gull_Weihai_115_2016_H13N2	Asia	Gull
MF461192_A_black-tailed_gull_Weihai_17_2016_H13N8	Asia	Gull
MH068339_A_laughing_gull_New_Jersey_UGAI17-2839_2017_H13N6	North America	Gull
MH134614_A_environment_Chile_C7144_2016_H13N2	South America	Environment
MH134699_A_environment_Chile_C7125_2016_H13N2	South America	Environment
MH488106_A_environment_Chile_C20076_2016_H13N9	South America	Environment
MH498659_A_Franklins_gull_Chile_C17421_2016_H13N9	South America	Gull
MH498705_A_kelp_gull_Chile_C8599_2016_H13N2	South America	Gull
MH498758_A_environment_Chile_C20182_2016_H13N9	South America	Environment
MH498788_A_Franklins_gull_Chile_C20069_2016_H13N9	South America	Gull
MH498819_A_black_skimmer_Chile_C20057_2016_H13N8	South America	Shorebird
MH498843_A_environment_Chile_C20177_2016_H13N9	South America	Environment
MH498926_A_Franklins_gull_Chile_C20094_2016_H13N9	South America	Gull
MH499086_A_American_oystercatcher_Chile_C20102_2016_H13N9	South America	Other Charadriiformes
MH499117_A_Franklins_gull_Chile_C20061_2016_H13N9	South America	Gull
MH499157_A_Franklins_gull_Chile_C20070_2016_H13N9	South America	Gull
MH499199_A_black_skimmer_Chile_C20142_2016_H13N9	South America	Shorebird
MH501034_A_gull_New_Jersey_UGAI15-3416_2015_H13N6	North America	Gull
MH501058_A_gull_New_Jersey_UGAI15-3767_2015_H13N3	North America	Gull
MH501661_A_ruddy_turnstone_New_Jersey_AI12-1737_2012_H13N6	North America	Shorebird
MH579143_A_kelp_gull_Delaware_Bay_304_2007_H13N9	North America	Gull
MH579242_A_semipalmated_sandpiper_Delaware_Bay_142_2007_H13N9	North America	Shorebird
MH596991_A_environment_New_Jersey_AI00-2413_2000_H13N6	North America	Environment
MH596999_A_herring_gull_New_Jersey_AI07-418_2007_H13N9	North America	Gull
MH733691_A_ruddy_turnstone_Delaware_Bay_204_2009_H13N6	North America	Shorebird
MH763863_A_ring-billed_gull_Minnesota_OPMNAI0816_2017_H13N8	North America	Gull
MH763998_A_ring-billed_gull_Minnesota_CLMNAI0891_2017_H13N6	North America	Gull
MH764092_A_ring-billed_gull_Minnesota_CLMNAI1199_2017_H13N2	North America	Gull
MH764225_A_ring-billed_gull_Minnesota_CLMNAI1301_2017_H13N2	North America	Gull
MH764241_A_ring-billed_gull_Minnesota_CLMNAI1501_2017_H13N2	North America	Gull
MH764249_A_ring-billed_gull_Minnesota_CLMNAI1529_2017_H13N6	North America	Gull
MK345841_A_laughing_gull_Delaware_Bay_451_2018_H13N6	North America	Gull
MK637077_A_herring_gull_Delaware_Bay_22_2014_H13N6	North America	Gull
MN210175_A_Glaucous-Winged_Gull_Southcentral_Alaska_17MB03625_2017_H13N2	North America	Gull
MN210183_A_Glaucous-Winged_Gull_Southcentral_Alaska_17MB03638_2017_H13N2	North America	Gull
MN210272_A_Herring_Gull_Massachusetts_A00648685_2008_H13N2	North America	Gull
MN783531_A_Red_Knot_Delaware_Bay_3_2019_H13N6	North America	Shorebird
MW126759_A_slender-billed_gull_Ukraine_CHBZ-11_2009_H13N2	Europe	Gull
MW132950_A_shelduck_Ukraine_KT-9-2-11_2016_H13N6	Europe	Anseriformes
MW873570_A_glaucous-winged_gull_Alaska_AK18-288_2018_H13N6	North America	Gull
MW874991_A_Red_Knot_Delaware_Bay_605_2020_H13N6	North America	Shorebird
MZ005199_A_Environmental_Santo_Domingo_GF89_2014_H13N2	South America	Environment
MZ015625_A_Glaucous-Winged_Gull_Alaska_20MB02182_2020_H13N6	North America	Gull
MZ015714_A_Glaucous-Winged_Gull_Alaska_20MB02820_2020_H13N6	North America	Gull
OL355047_A_Kelp_gull_Peru_M8_2019_H13N6	South America	Gull
OL369952_A_silver_gull_Tasmania_06-0349-105_2006_H13N6	Oceania	Gull
OM965830_A_Red_Knot_Delaware_224_2021_H13N6	North America	Shorebird
OP888545_A_kelp_gull_Chile_C25097_2017_H13N9	South America	Gull
OP888639_A_environment_Chile_C39659_2018_H13N2	South America	Environment
OP888815_A_environment_Chile_C39624_2018_H13N2	South America	Environment
OP888838_A_southern_lapwing_Chile_C36480_2018_H13N8	South America	Other Charadriiformes
OP888900_A_Franklins_gull_Chile_C33636_2017_H13N2	South America	Gull
OP888964_A_yellow-billed_teal_Chile_C38424_2018_H13N8	South America	Anseriformes
OQ366681_A_Laughing_Gull_DE_801_2022_H13N2	North America	Gull
OQ366759_A_Laughing_Gull_DE_827_2022_H13N2	North America	Gull

OQ368516_A_Red_Knot_DE_1087_2022_H13N6	North America	Shorebird
OQ691624_A_Franklins_gull_USA_D21-004347-2_MNAI1144_2017_H13N2	North America	Gull
SKH13N6_NS	SKH13N6	Gull
U96738_A_gull_Minnesota_945_1980_H13N6	North America	Gull
